# MHC-II deficiency in a large Algerian cohort: Clinical, immunological, and genetic characterization

**DOI:** 10.70962/jhi.20260162

**Published:** 2026-10-30

**Authors:** Lydia Lamara Mahammed, Brahim Belaid, Koon-Wing Chan, Aida Mohand-oussaid, Ouardia Drali, Abdelhak Dehimi, Daniel Leung, Lilya Meriem Berkani, Hassiba Sakhri, Asmaa Abed, Meriem Tahir, Fatma Merah, Amina Salmi, Wassila Messadi, Souhila Melzi, Jing Yang, Xing Tian Yang, Jennifer Stoddard, Julie Niemela, Heather Kenney, Fella Dahmoune, Fairouz Guerrouche, Ahlem Mechali, Amina Saad Djaballah, Lamia Amirat, Meriem Oussalah, Yacine Inouri, Saadeddine Dib, Fatima Benbida, Chahynez Boubidi, Hind Ismahen Titri, Lynda Sekfali, Sidi Mohamed Azzouz, Amel Laredj, Fadela Bendaoud, Halima Zerguine, Djohra Hadef, Ahmed Kadi, Djazia Elhadef, Hamza Boucenna, Khadidja Benallal, Moufida Djalab, Chahrazed El Mezouar, Rachid Bouhdjila, Mimouna Bessahraoui, Nadia Bensaadi, Farih Mehdid, Malek Benakli, Farida Harieche, Rawda Aboura, Zoulikha Zeroual, Leila Smati, Salih Bendeddouche, Reda Belbouab, Azzeddine Mekki, Belkacem Bioud, Samir Mohamed Ladj, Merzak Gharnaout, Asmahane Ladjouze, Ourida Gacem, Nafissa Benhalla, Jaime Sou Rosa Duque, Sergio Rosenzweig, Luigi Daniele Notarangelo, Yu Lung Lau, Reda Djidjik

**Affiliations:** 1 Department of Immunology, Beni Messous University Hospital Center, Algiers Faculty of Pharmacy, The University of Health Sciences, Algiers, Algeria; 2 Department of Pediatrics and Adolescent Medicine, School of Clinical Medicine, Li Ka Shing Faculty of Medicine, The University of Hong Kong, Hong Kong SAR, China; 3 Department of Pediatrics A, Beni Messous University Hospital Center, Algiers Faculty of Medicine, The University of Health Sciences, Algiers, Algeria; 4 Department of Pediatric Pulmonology and Allergology, Canastel Children’s Hospital, Oran Faculty of Medicine, The University of Oran, Oran, Algeria; 5 Department of Pediatrics, Setif University Hospital Center, Setif Faculty of Medicine, The University of Setif 1, Setif, Algeria; 6 Department of Immunology, Beni Messous University Hospital Center, Algiers, Algeria; 7 Department of Pediatrics B, Beni Messous University Hospital Center, Algiers Faculty of Medicine, The University of Health Sciences, Algiers, Algeria; 8 Department of Pediatrics, Bab El Oued University Hospital Center, Algiers Faculty of Medicine, The University of Health Sciences, Algiers, Algeria; 9 Immunology Service, Department of Laboratory Medicine, Clinical Center, National Institutes of Health, Bethesda, MD, USA; 10 National Institute of Allergy and Infectious Diseases, National Institutes of Health, Bethesda, MD, USA; 11 Department of Pediatrics, Bejaia University Hospital Center, Bejaia Faculty of Medicine, The University of Bejaia, Bejaia, Algeria; 12 Department of Pediatrics B, Hussein Dey University Hospital Center, Algiers Faculty of Medicine, The University of Health Sciences, Algiers, Algeria; 13 Department of Pediatrics B, Hussein Dey University Hospital Center, Algiers, Algeria; 14 Department of Pediatrics, Bologhine Hospital, Algiers Faculty of Medicine, The University of Health Sciences, Algiers, Algeria; 15 Department of Pediatrics, Birtraria Hospital, Algiers Faculty of Medicine, The University of Health Sciences, Algiers, Algeria; 16 Department of Pediatrics, Beni Messous Military Mother and Child Hospital, Algiers Faculty of Medicine, The University of Health Sciences, Algiers, Algeria; 17 Department of Pediatrics, Specialized Mother and Child Hospital of Tlemcen, Tlemcen Faculty of Medicine, The University of Tlemcen, Tlemcen, Algeria; 18 Department of Pediatrics, Specialized Mother and Child Hospital of Tlemcen, Tlemcen, Algeria; 19 Department of Pediatrics A, Hussein Dey University Hospital Center, Algiers Faculty of Medicine, The University of Health Sciences, Algiers, Algeria; 20 Department of Pediatrics, Mustapha Bacha University Hospital Center, Algiers, Algeria; 21 Department of Pediatrics, Mustapha Bacha University Hospital Center, Algiers Faculty of Medicine, The University of Health Sciences, Algiers, Algeria; 22 Department of Pediatric Gastroenterology and Nutrition, Canastel Children’s Hospital, Oran Faculty of Medicine, The University of Oran, Oran, Algeria; 23 Department of Pediatric Infectious Diseases, Canastel Children’s Hospital, Oran Faculty of Medicine, The University of Oran, Oran, Algeria; 24 Department of Neonatology, Specialized Mother and Child Hospital of Batna, Batna Faculty of Medicine, The University of Batna, Batna, Algeria; 25 Department of Pediatrics, Specialized Mother and Child Hospital of Batna, Batna Faculty of Medicine, The University of Batna, Batna, Algeria; 26 Department of Pediatrics, Batna University Hospital Center, Batna Faculty of Medicine, The University of Batna, Batna, Algeria; 27 Department of Phthisiology and Pulmonology, Beni Messous University Hospital Center, Algiers Faculty of Medicine, The University of Health Sciences, Algiers, Algeria; 28 Department of Pediatrics, Sidi Bel Abbes University Hospital Center, Sidi Bel Abbes Faculty of Medicine, The University of Sidi Bel Abbes, Sidi Bel Abbes, Algeria; 29 Department of Pediatrics, Regional Military University Hospital of Constantine, Constantine Faculty of Medicine, The University of Constantine, Constantine, Algeria; 30 Department of Pediatrics B, Constantine University Hospital Center, Constantine Faculty of Medicine, The University of Constantine, Constantine, Algeria; 31 Department of Pediatrics, Tizi Ouzou University Hospital Center, Tizi Ouzou Faculty of Medicine, The University of Tizi Ouzou, Tizi Ouzou, Algeria; 32 Department of Hematology and Bone Marrow Transplantation, Pierre and Marie Curie Center, Algiers Faculty of Medicine, The University of Health Sciences, Algiers, Algeria; 33 Department of Hematology and Bone Marrow Transplantation, Pierre and Marie Curie Center, Algiers Faculty of Pharmacy, The University of Health Sciences, Algiers, Algeria; 34 Department of Pediatrics, Tlemcen University Hospital Center, Tlemcen Faculty of Medicine, The University of Tlemcen, Tlemcen, Algeria

## Abstract

MHC class II (MHC-II) deficiency is a rare inborn error of immunity caused by defects in transcriptional regulators of MHC-II expression. In this study, we characterized the clinical, immunological, and molecular features of 107 patients from 98 unrelated Algerian families. Median ages at symptom onset and diagnosis were 5 and 14 mo, respectively, with pneumonia (82%) and chronic diarrhea (73%) predominating. CD4^+^ T cell lymphopenia occurred in 87.6% and naïve CD4^+^ T cell depletion in 86.0%. Among 99 genetically characterized patients, variants were identified exclusively in *RFXANK* (77.8%) and *RFXAP* (22.2%), including two predominant mutations (*RFXANK* c.338-25_338del and *RFXAP* c.368del) and four novel variants. Exploratory comparisons suggested a more severe phenotype in patients carrying *RFXAP* c.368del than in those carrying *RFXANK* c.338-25_338del. Five of seven transplanted patients survived versus 33.3% of non-transplanted patients. As the largest MHC-II deficiency cohort reported to date, this study substantially expands current knowledge of the disease and supports targeted genetic diagnosis in North African populations.

## Teaser

This study characterizes the clinical, immunological, and genetic spectrum of MHC-II deficiency in the largest cohort reported to date. It identifies recurrent and novel variants and reveals genotype–phenotype differences.

## Introduction

MHC class II (MHC-II) deficiency is a rare autosomal recessive inborn error of immunity (IEI) first described in the 1980s (1, 2, 3). Although cases from diverse ethnic backgrounds have been reported worldwide, a marked predominance is observed in the Middle East and North Africa (MENA), where more than 700 patients have been reported (4). Notably, a particularly high frequency is observed in the Maghreb region (Algeria, Morocco, and Tunisia) (5, 6, 7, 8, 9), likely reflecting regional founder effects and high rates of consanguinity.

The molecular basis of MHC-II deficiency involves pathogenic variants affecting one of the four genes that encode transcriptional regulators required for MHC-II expression: regulatory factor X–associated protein containing ankyrin repeat (*RFXANK*), regulatory factor X-5 (*RFX5*), regulatory factor–associated protein (*RFXAP*), and class II trans-activator (*CIITA*) (10). These defects lead to absent or markedly reduced MHC-II expression on antigen-presenting cells, resulting in impaired CD4^+^ T cell development and activation, as well as impaired T helper–dependent antibody responses, ultimately compromising both cellular and humoral immunity (10).

The resulting combined immunodeficiency (CID) leads to recurrent and often life-threatening infections, predominantly affecting the respiratory and gastrointestinal tracts, and frequently associated with failure to thrive (7, 11, 12, 13). In most patients, symptoms arise within the first year of life; however, milder phenotypes have been reported, with diagnosis delayed until adolescence in some cases (14). The prognosis remains poor in the absence of curative treatment, and hematopoietic stem cell transplantation (HSCT) currently represents the only definitive therapeutic option, but results are less favorable compared with other combined immune deficiencies (15, 16).

Immunologically, MHC-II deficiency is characterized by absent or markedly reduced MHC-II expression, often associated with hypogammaglobulinemia, CD4^+^ T cell lymphopenia, and impaired antigen-specific cellular responses. In the absence of a genetic diagnosis, the combination of these clinical and laboratory characteristics strongly suggests MHC-II deficiency, in accordance with the current European Society for Immunodeficiencies (ESID) diagnostic criteria (17).

In Algeria, MHC-II deficiency ranks as the third most frequent disorder among IEIs and the second most common form of CID (18). Algerian cases have been included in several reports since the early descriptions of the disease (5, 7, 8, 19). The founder *RFXANK* c.338-25_338del (formerly c.752delG26) mutation was the most commonly reported molecular defect in these patients (5, 7, 8). Nevertheless, there have been no dedicated reports from Algeria following the 2012 study by Djidjik et al. (8), leaving a major gap in defining the national disease spectrum.

In this context, the present study aimed to provide a comprehensive characterization of the clinical, immunological, and genetic features of the largest MHC-II deficiency cohort reported to date, comprising 107 patients, 92.5% of whom were genetically characterized, thereby substantially expanding the currently available data on this rare disorder.

## Results

### Patient characteristics and clinical features

**Figure 1.**
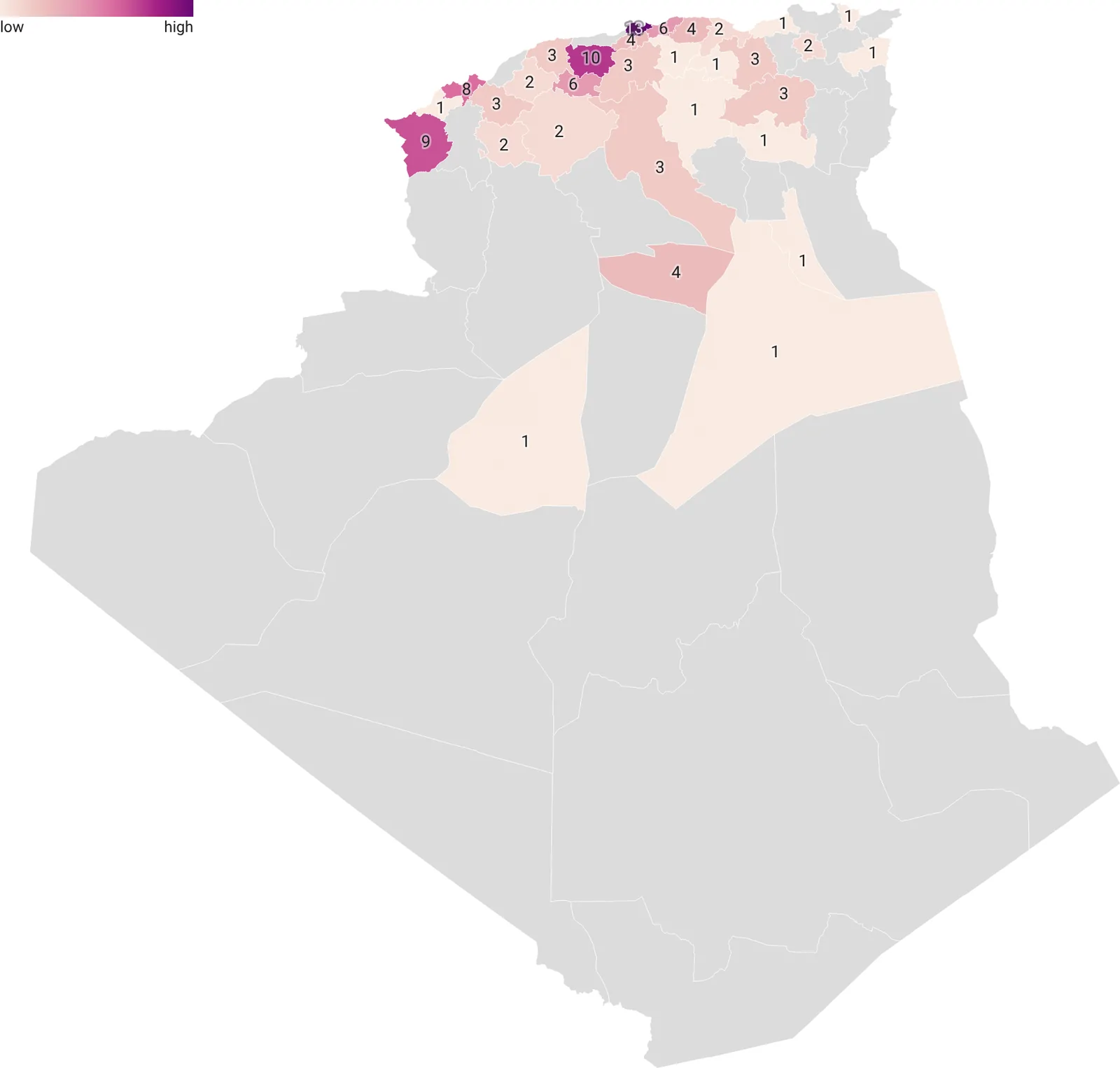
**Geographic distribution of patients with MHC-II deficiency across Algerian provinces.** Numbers on the map represent patient counts per province; grey areas represent regions with no patients in the cohort. Data were available for 103 patients.

A total of 107 patients from 98 unrelated families were included in this study, comprising 65 males and 42 females (Fig. 1, Table S1, and Table 1). The median ages at onset and diagnosis were 5 mo (range, 0–72) and 14 mo (range, 0–168), respectively, corresponding to a median diagnostic delay of 9 mo (interquartile range [IQR]: 3–19). Consanguinity was reported in 60.7% of cases. 10 patients had a sibling with a confirmed diagnosis of MHC-II deficiency, and 37 had one or more siblings who died at a young age with a clinical phenotype suggestive of MHC-II deficiency. Overall, patients with a positive family history were diagnosed at a significantly younger age than those without a family history (median age at diagnosis, 9 versus 17.5 mo; P = 0.001).

**Table 1.** Demographic and clinical characteristics of patients

Characteristics	*N* = 107
Consanguinity, *n* (%)	65 (60.7)
Family history, *n* (%)	47 (43.9)
Sex, male/female (ratio)	65/42 (1.5)
Age at onset (months), median (Q1, Q3)	5 (3, 9)
Age at diagnosis (months), median (Q1, Q3)	14 (6, 32)
Diagnostic delay (months), median (Q1, Q3)	9 (3, 19)
**Clinical presentation on admission** ^a^ **, *n* (%)**	
Recurrent pneumonia	82 (82)
Chronic diarrhea	73 (73)
ENT recurrent infections	35 (35)
Failure to thrive	34 (34)
Oral thrush	29 (29)
Urinary tract infections	17 (17)
Cutaneous manifestations	17 (17)
—Skin infections	12/17 (70.5)
—Eczema	2/17 (11.8)
—Maculopapular rash	2/17 (11.8)
—Erythematovesicular lesions	1/17 (5.9)
Neurological features	12 (12)
—Nystagmus	5/12 (41.7)
—Inflammatory central nervous system disorders	4/12 (33.3)
—Psychomotor developmental delay	2/12 (16.7)
—Hypotonia	2/12 (16.7)
—Seizures	1/12 (8.3)
Fungal gastrointestinal infections	8 (8)
Lymphoproliferative manifestations	7 (7)
—Lymphadenopathy	5/7 (71.4)
—Splenomegaly	2/7 (28.6)
—Hepatomegaly	1/7 (14.3)
Septicemia	6 (6)
Autoimmunity	5 (5)
—Autoimmune thyroiditis	2/5 (40)
—Autoimmune hemolytic anemia	2/5 (40)
—Thrombocytopenia	1/5 (20)
Allergic manifestations	3 (3)
—Allergic asthma	2/3 (66.7)
—Cow’s milk protein allergy	1/3 (33.3)
Inflammatory gastrointestinal manifestations	2 (2)
Cholangiectasis	1 (1)

a Clinical data were restricted to 100 symptomatic patients, excluding seven asymptomatic cases screened during the neonatal period because of a familial history of the disease. Subcategory percentages were calculated using the number of patients within the corresponding clinical category as the denominator. Patients could present with more than one manifestation. ENT, ear, nose, and throat.

Except for seven patients who were screened during the neonatal period, the remaining 100 symptomatic patients had one or more clinical manifestations at admission (Table 1). The clinical presentation was dominated by recurrent pneumonia (82%), chronic diarrhea (73%), recurrent ear, nose, and throat infections (35%), and failure to thrive (34%).

Cutaneous involvement was documented in 17 patients (17%), including skin infections (12/17), eczema (2/17), maculopapular rash (2/17), and erythematovesicular lesions (1/17).

Neurological manifestations were reported in 12 patients (12%). Nystagmus was the most common neurological feature (5/12), followed by inflammatory disorders of the central nervous system (4/12), including two cases of meningoencephalitis, one of meningitis, and one of encephalitis. Psychomotor developmental delay (2/12) and hypotonia (2/12) were also reported, whereas seizures occurred in one patient.

Lymphoproliferative manifestations were documented in seven patients (7%), predominantly lymphadenopathy (5/7), followed by splenomegaly (2/7) and hepatomegaly (1/7). Five patients presented autoimmune diseases, including autoimmune thyroiditis (2/5), autoimmune hemolytic anemia (2/5), and immune thrombocytopenia (1/5). Allergic manifestations were observed in three patients (3%), including allergic asthma (*n* = 2) and cow’s milk protein allergy (*n* = 1).

Gastrointestinal manifestations extended beyond chronic diarrhea and comprised fungal infections in 8% of patients, mainly digestive candidiasis and *Candida* esophagitis, as well as inflammatory complications in 2%, including colitis, perianal abscess, and anal ulceration. Hepatobiliary involvement was documented in one patient (1%), who had a *Cryptosporidium* infection associated with cholangiectasis.

### Immunological findings

**Figure 2.**
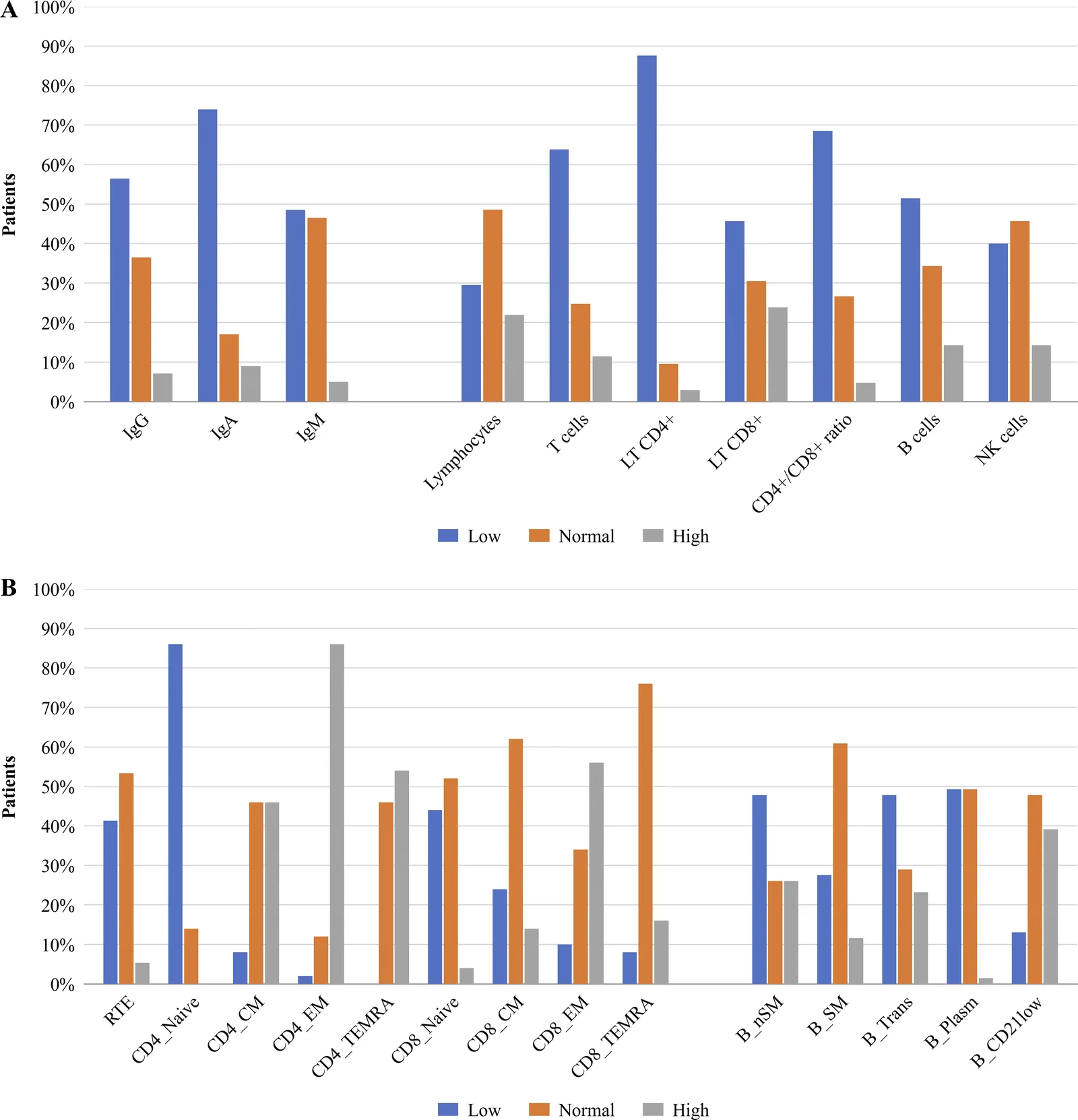
**Immunological profile of patients with MHC class II deficiency** **.** **(A)** Distribution of immunoglobulin levels and major lymphocyte subsets. **(B)** Distribution of T- and B-cell subsets. The panels show the proportions of patients with decreased, normal, or increased values for each parameter according to age-matched reference ranges. Denominators indicate available data for each parameter: IgG (*n* = 85), IgA (*n* = 100), IgM (*n* = 101), total lymphocytes/T/B/NK cells (*n* = 105), RTE (*n* = 75), T-cell subsets (*n* = 50), and B-cell subsets (*n* = 69). CM, central memory; EM, effector memory; Ig, immunoglobulins; NK, natural killer; nSM, non-switched memory; Plasm, plasmablasts; RTE, recent thymic emigrant; TEMRA, terminally differentiated effector memory re-expressing CD45RA; Trans, transitional; SM, switched memory.

**Figure S1.**
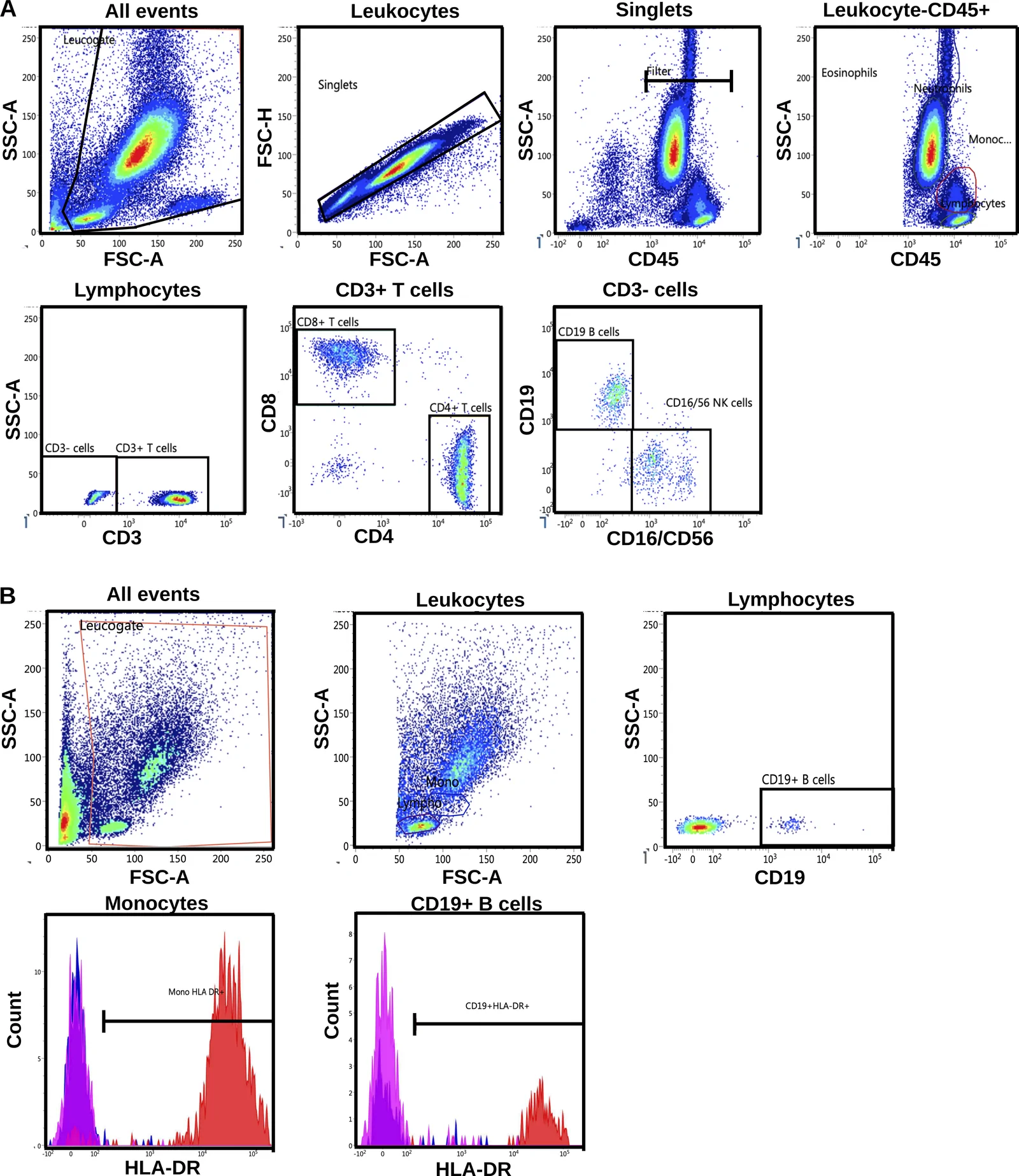
**Gating strategy for major lymphocyte subsets and assessment of HLA-DR expression. (A)** Main lymphocyte subsets: T, B, NK cells. **(B)** HLA-DR expression on monocytes and CD19^+^ B cells is shown for the patient (blue), the isotype control (pink), and a healthy control (red).

**Figure S2.**
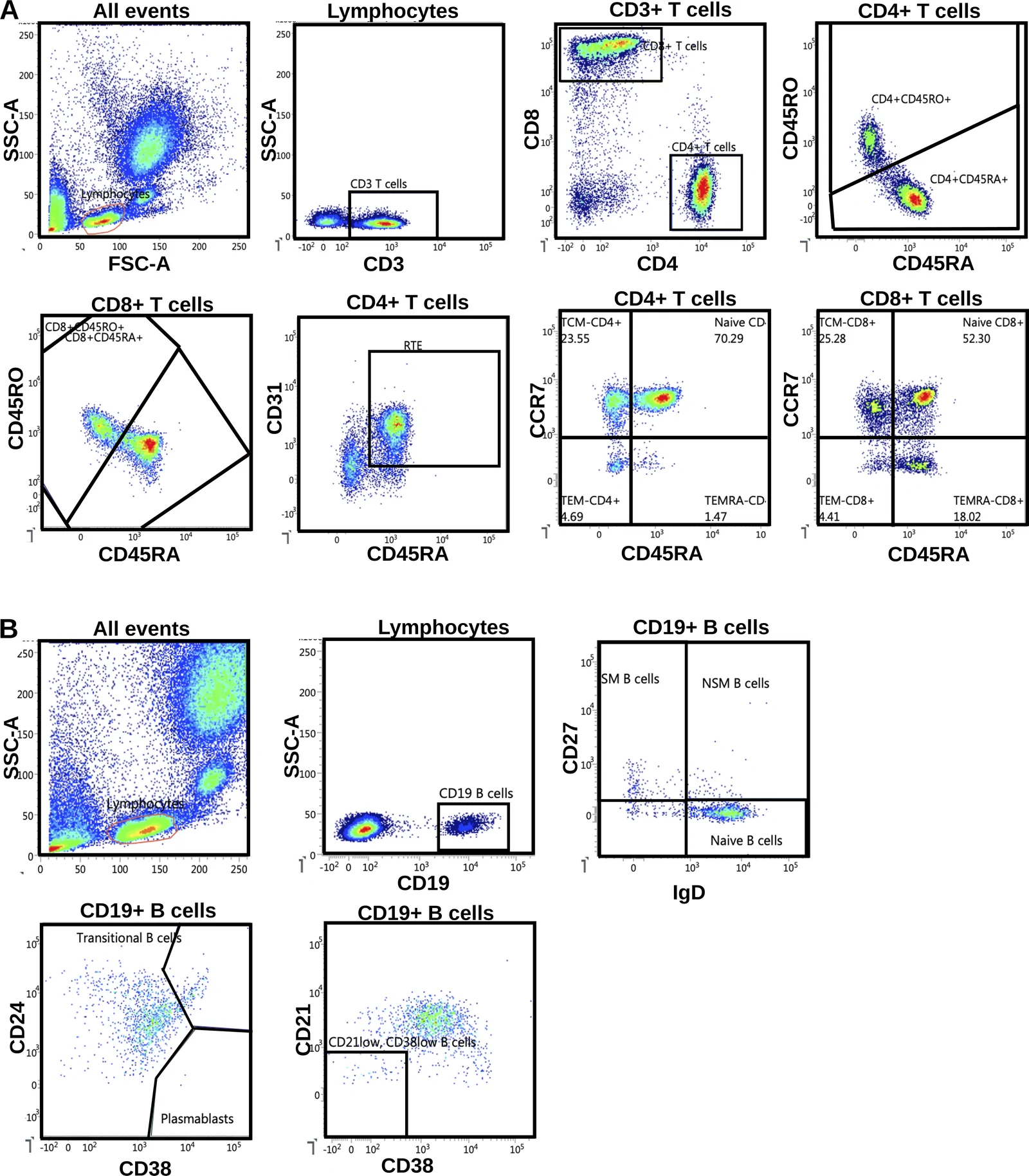
**Gating strategy for extended T and B cell immunophenotyping. (A)** Extended T cell immunophenotyping. **(B)** Extended B cell immunophenotyping.

Marked abnormalities were observed across both humoral and cellular compartments (Fig. 2). Reduced Ig levels were most frequent for IgA (74/100, 74.0%), followed by IgG (48/85, 56.5%) and IgM (49/101, 48.5%). Regarding HLA-DR expression, all included patients showed complete absence of HLA-DR expression, with no residual expression detected. Further immunophenotyping, performed using the antibody panels and gating strategies presented in Table S2, Fig. S1, and Fig. S2, respectively, revealed low total T cell counts in 63.8% (67/105) of patients and a particularly pronounced reduction in CD4^+^ T cell counts, detected in 87.6% (92/105) of cases, alongside a reversed CD4^+^/CD8^+^ T cell ratio in 68.6% (72/105). B cell lymphopenia and reduced natural killer (NK) cell counts were also observed in a non-negligible proportion of patients, affecting 51.4% (54/105) and 40.0% (42/105) of patients, respectively (Fig. 2 A).

Extended phenotyping revealed a profound disturbance of T cell maturation, characterized by depletion of naïve CD4^+^ T cells (43/50, 86.0%) with expansion of differentiated compartments, including CD4^+^ effector memory T cells (EM) (43/50, 86.0%) and terminally differentiated EM re-expressing CD45RA (TEMRA) (27/50, 54.0%). Similar but less pronounced skewing was observed in the CD8^+^ compartment, with reduced naïve CD8^+^ cells (22/50, 44.0%) and expanded EM cells (28/50, 56.0%) (Fig. 2 B).

B cell subset abnormalities included frequent reductions in the percentages (from B cells) of non-switched memory B cells (nSMs; 33/69, 47.8%), transitional B cells (33/69, 47.8%), and plasmablasts (34/69, 49.3%), whereas the proportion of switched memory B cells (SMs) was within the normal range in most patients (42/69, 60.9%). An expansion of the CD21^–/low^ B cell subset, associated with B cell autoreactivity, was observed in 39.1% (27/69) of cases (Fig. 2 B). In an exploratory analysis, among the 65 patients for whom CD21^–/low^ B cell frequencies and clinical data were available, autoimmune manifestations were observed in 4 of 27 patients with increased CD21^–/low^ B cell frequencies (14.8%), whereas none of the 38 patients without increased frequencies had autoimmune manifestations (Fisher’s exact test, P = 0.026).

### Mutation analysis

**Figure 3.**
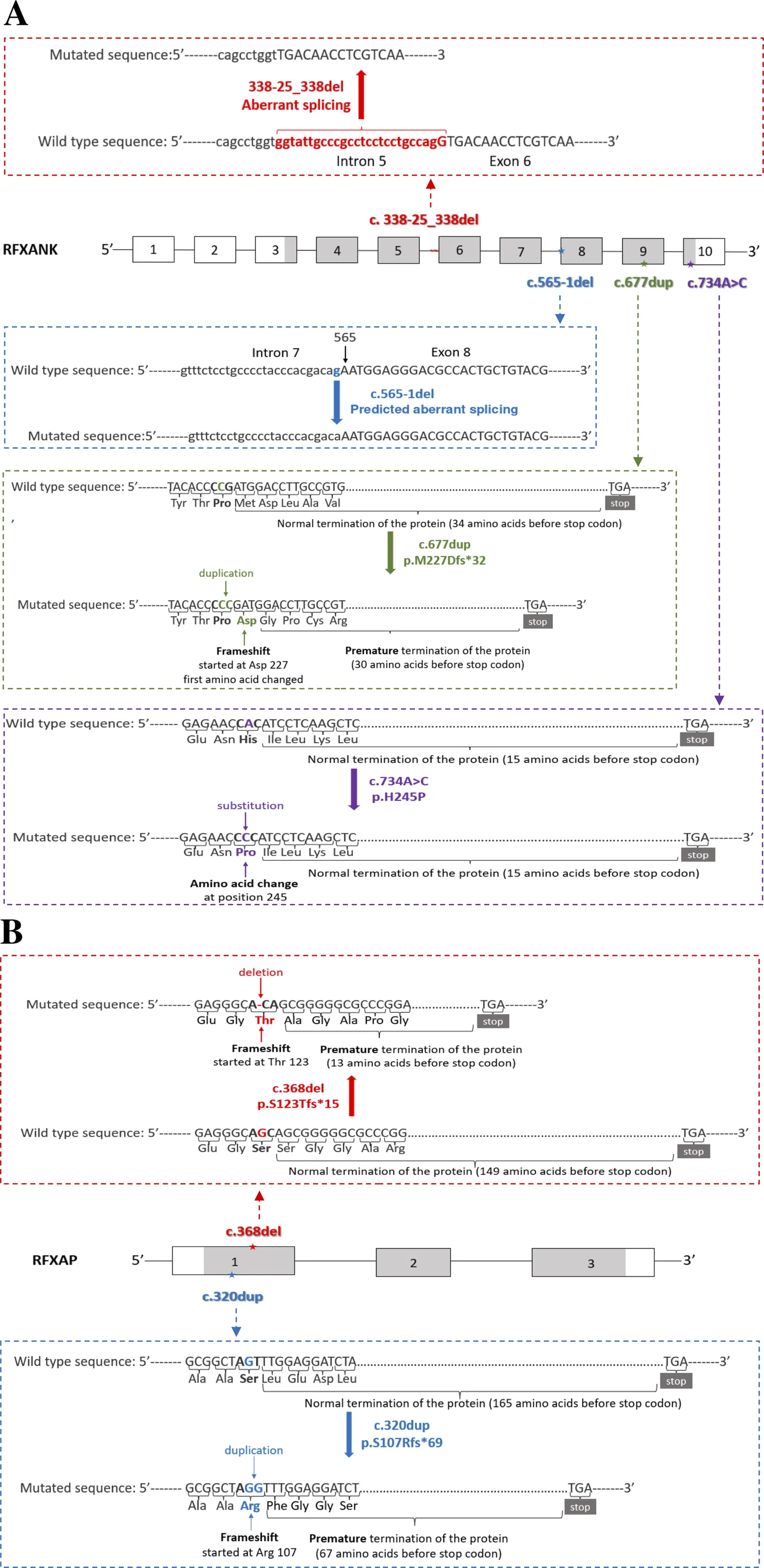
**Detailed representation of *RFXANK* and *RFXAP* variants identified in the present study. (A)** Schematic representation of *RFXANK* variants. The previously reported mutation (c.338-25_338del) is shown at the top, whereas the three novel variants (c.565-1del, c.677dup and c.734A>C) are shown below. **(B)** Schematic representation of *RFXAP* variants. The previously reported mutation (c.368del) is shown at the top, whereas the novel variant (c.320dup) is shown below.

Genetic analysis was performed in 99 patients. 77 patients (77.8% of the tested cases) had mutations in the *RFXANK* gene, and 22 (22.2%) had mutations in the *RFXAP* gene (Fig. 3). A total of six distinct variants were identified in our cohort (Table 2), all in the homozygous state.

**Table 2.** Summary of the genetic mutations

Gene	Nucleotide change (HGVS)	Protein change	Variant type	Inheritance	Variant characteristics	Carriers, *n*
*RFXANK*	c.338-25_338del	/	Splice	Homozygous	Reported pathogenic	71
*RFXANK*	c.565-1del	/	Splice	Homozygous	**Novel** ACMG: pathogenic Splice AI Δ score: 0.97 for acceptor loss	3
*RFXANK*	c.677dup	p. Met227AspfsTer32	Frameshift duplication	Homozygous	**Novel** ACMG: pathogenic	2
*RFXANK*	c.734A>C	p.His245Pro	Missense	Homozygous	**Novel** ACMG: likely pathogenic PolyPhen-2 score: 1 SIFT score: 0 CADD PHRED score: 28.1 REVEL: 0.464	1
*RFXAP*	c.368del	p.Ser123ThrfsTer15	Frameshift deletion	Homozygous	Reported pathogenic	21
*RFXAP*	c.320dup	p.Ser107ArgfsTer69	Frameshift duplication	Homozygous	**Novel** ACMG: pathogenic	1

**Figure S3.**
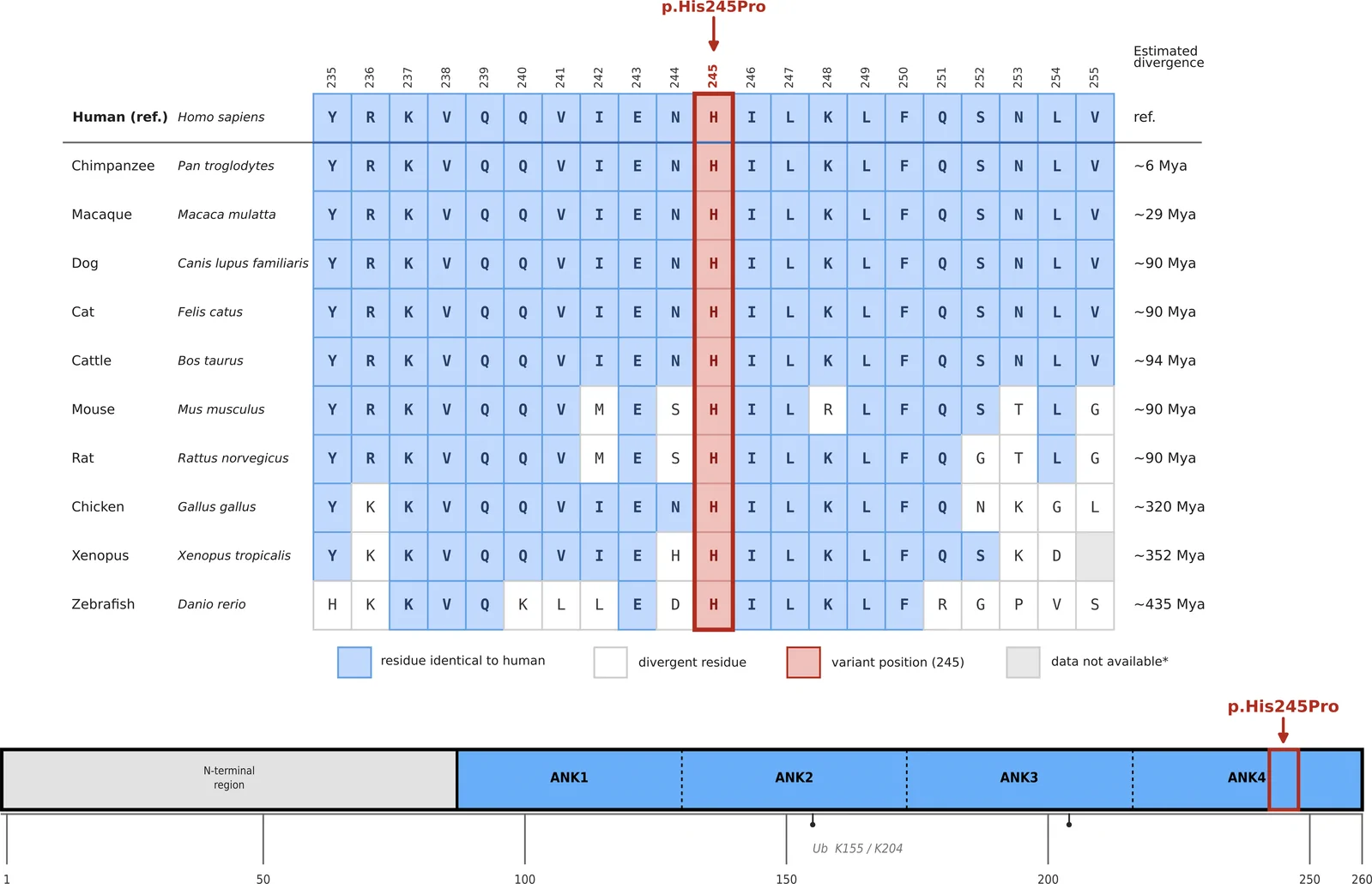
**Conservation of the RFXANK p.His245 residue across vertebrates and domain structure of RFXANK (260 aa) and position of p.His245.** *Xenopus tropicalis (XP_002936373.2): deposited isoform sequence truncated downstream of position 254.

Regarding the *RFXANK* gene (Fig. 3 A), 71 cases carried the previously reported deletion c.338-25_338del, representing 71.7% of the tested patients and 92.2% of those carrying an *RFXANK* mutation, while six patients harbored novel mutations. Three patients, from three unrelated families, presented with a single-base-pair deletion in intron 7 (c.565-1del), predicted to affect the invariant AG acceptor splice site of exon 8 and to result in aberrant splicing (Splice AI Δ score: 0.97 for acceptor loss). Two patients, from two unrelated families, carried a single-nucleotide duplication at position 677 in exon 9 (c.677dup) predicted to cause a frameshift resulting in a premature truncation of the protein 32 aa’s downstream of codon 227 (p.Met227AspfsTer32). The last case harbored a missense mutation in exon 10 (c.734A>C) changing the aa at position 245 (p.His245Pro). The affected histidine residue is highly conserved across vertebrate species and is located within the fourth ankyrin domain (Fig. S3). Most in silico prediction tools suggested a deleterious effect of this variant (PolyPhen-2 score: 1; Sorting Intolerant From Tolerant [SIFT] score: 0; Combined Annotation Dependent Depletion [CADD] -PHRED score: 28.1), although the Rare Exome Variant Ensemble Learner (REVEL) score indicated a moderate pathogenicity (0.464).

In addition to mutations in *RFXANK*, two variants in the *RFXAP* gene were identified (Fig. 3 B). 21 cases carried the previously reported deletion c.368del (p.Ser123ThrfsTer15), representing 21.2% of the tested patients and 95.5% of those carrying an *RFXAP* mutation. The remaining case harbored a novel mutation: c.320 dup. This single-nucleotide duplication at position 320 in exon 1 was predicted to cause a frameshift resulting in a premature truncation of the protein 69 aa’s downstream of codon 107 (p.Ser107ArgfsTer69).

**Figure S4.**
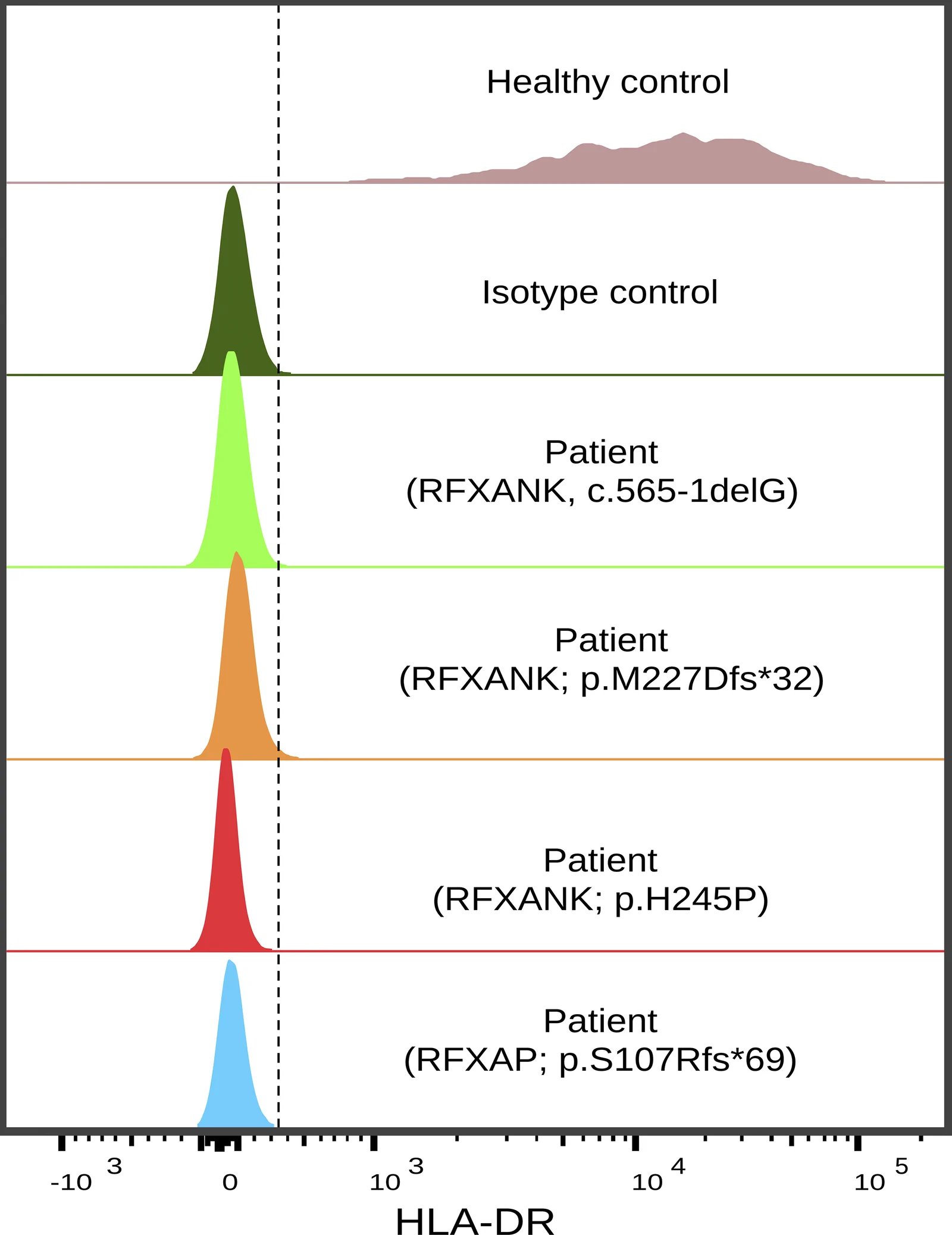
HLA-DR expression on B cells in patients carrying novel variants.

Flow cytometric analysis of B cells from patients carrying the four novel variants demonstrated absent HLA-DR surface expression (Fig. S4). Notably, all four variants were identified by whole-exome sequencing (WES) and confirmed by targeted Sanger sequencing.

### Outcomes and follow-up

**Figure 4.**
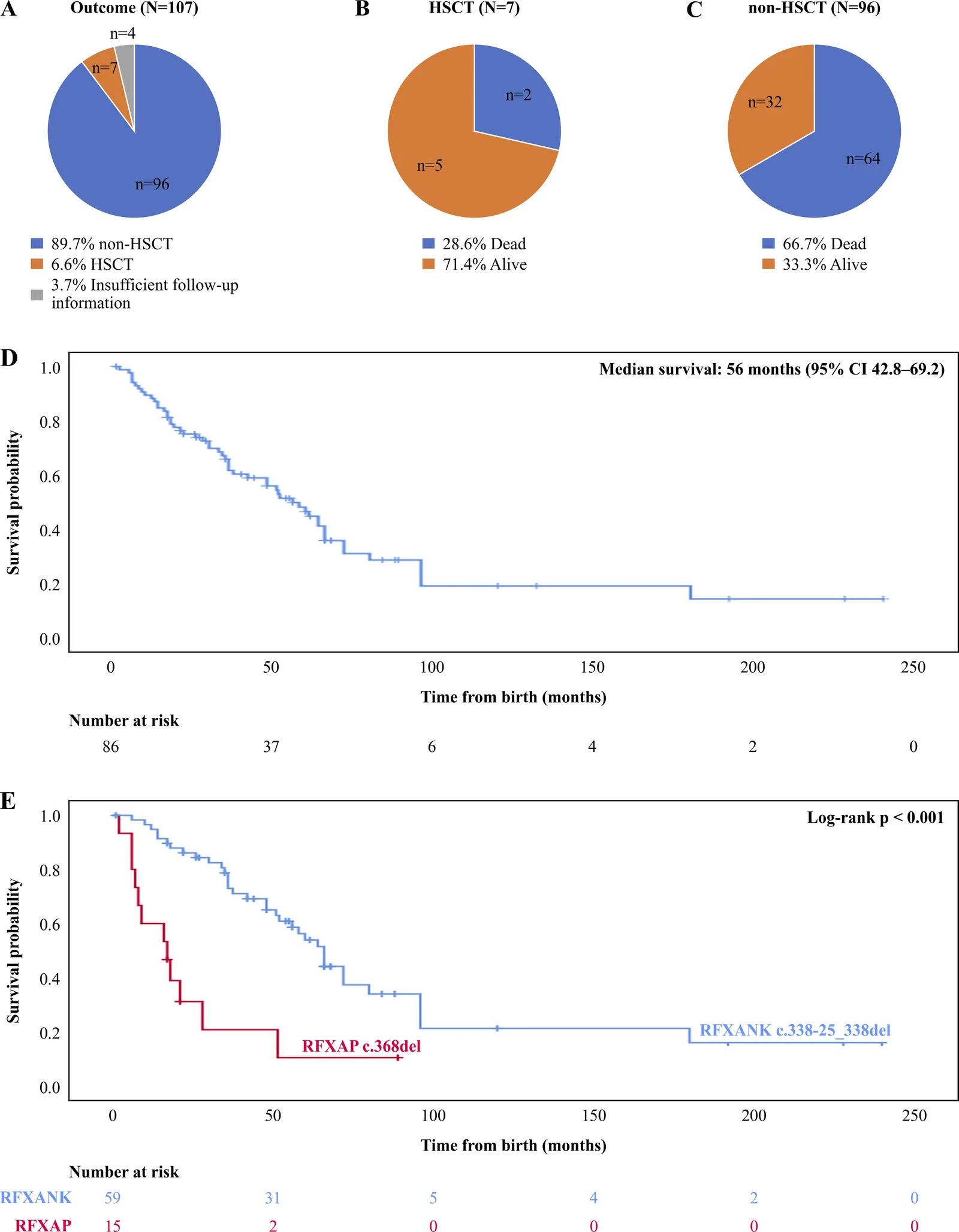
**Clinical outcomes and survival of patients with MHC-II deficiency. (A)** Distribution of patients according to transplantation status. **(B)** Outcome of transplanted patients. **(C)** Outcome of non-transplanted patients. **(D)** Kaplan–Meier survival curve of non-transplanted patients with available time-to-event data (*N* = 86). **(E)** Kaplan–Meier survival curves according to genotype (*RFXANK* c.338-25_338del versus *RFXAP* c.368del) among non-transplanted patients with available time-to-event data (*N* = 74 [*RFXANK* group *n* = 59; *RFXAP* group *n* = 15]).

Of the 107 patients, 96 (89.7%) did not undergo HSCT, 7 (6.6%) underwent HSCT, and 4 (3.7%) had insufficient follow-up information to determine their transplantation and vital status (Fig. 4 A).

At the last follow-up, five of the seven transplanted patients (71.4%) were alive (Fig. 4 B and Table S3), and among the 96 non-transplanted patients, 32 (33.3%) were alive (Fig. 4 C).

For the Kaplan–Meier analysis, 10 deceased non-transplanted patients were excluded because their age at death was unavailable. The analysis therefore included 86 patients, comprising 54 deaths and 32 censored observations, and estimated a median survival from birth of 56 mo (95% confidence interval [CI]: 42.8–69.2 mo) (Fig. 4 D).

Among the seven transplanted patients, five underwent HSCT in Algeria at a median age of 6.4 years (range, 4.5–10 years). HLA-DR expression was assessed in four patients and was restored in all four, including one evaluated 8 mo after HSCT. Comprehensive immunological assessment was available for three patients after a median follow-up of 30 mo (range, 29–32 mo). Total CD3^+^ T cell counts were within or above the normal range, whereas CD4^+^ T cell counts were within the normal range in two patients but remained reduced in one. In contrast, naïve CD4^+^ T cell reconstitution remained incomplete in all three patients. Detailed posttransplant immunological findings are provided in Table S3.

### Genotype–phenotype comparison between patients carrying the *RFXANK* c.338-25_338del and *RFXAP* c.368del mutations

Comparative analysis of patients carrying the two most frequent mutations revealed significant differences in both clinical and immunological features (Table 3). Patients harboring the *RFXANK* c.338-25_338del mutation showed significantly higher median ages at symptom onset and diagnosis than those carrying *RFXAP* c.368del (P = 0.002 and P < 0.001, respectively). Clinically, ENT infections were significantly more prevalent among patients with *RFXANK* c.338-25_338del, whereas neurological manifestations were observed more frequently in those with *RFXAP* c.368del. Notably, nystagmus, the most common neurological feature in the cohort, was observed exclusively in patients with the *RFXAP* c.368del mutation (5/20 versus 0/68).

**Table 3.** Comparative analysis of patients with *RFXANK* c.338-25_338del mutation and those with the *RFXAP* c.368del mutation

Characteristics	*RFXANK* c.338-25_338del (*n* = 71)	*RFXAP* c.368del (*n* = 21)	P
Age at onset (months), median (Q1–Q3)	6 (3.8–12)	3 (2–5)	**0.002**
Age at diagnosis (months), median (Q1–Q3)	21 (11.5–48)	6 (5–8)	**<0.001**
Calendar year of diagnosis, median (IQR)	2021 (2017–2023)	2022 (2020–2023)	0.246
**Clinical presentation on admission** ^a^ **, *n* (%)**			
Recurrent pneumonia	53 (77.9)	18 (90)	0.339
Persistent diarrhea	51 (75)	13 (65)	0.377
ENT recurrent infections	28 (41.2)	2 (10)	**0.014**
Failure to thrive	27 (39.7)	6 (30)	0.431
Oral thrush	18 (26.5)	6 (30)	0.755
Urinary tract infections	14 (20.6)	3 (15)	0.752
Neurological features	5 (7.4)	7 (35)	**0.002**
Skin infections	11 (16.2)	0 (0)	0.063
Fungal gastrointestinal infections	6 (8.8)	1 (5)	1
Lymphoproliferation	5 (7.4)	0 (0)	0.584
Septicemia	5 (7.4)	1 (5)	1
Autoimmunity	5 (7.4)	0 (0)	0.584
Non-infectious cutaneous manifestations	4 (5.9)	1 (5)	1
Allergic manifestations	2 (2.9)	1 (5)	0.543
Inflammatory gastrointestinal manifestations	2 (2.9)	0 (0)	1
**Immunological assessment** ^b^	​	​	​
IgG (g/L), median (Q1–Q3)	3.3 (1.2–6)	0.7 (0.4–1.7)	**<0.001**
Low IgG, *n*/*N* (%)	28/53 (52.8)	15/20 (75)	0.086
IgA (g/L), median (Q1–Q3)	0.1 (0.06–0.26)	0.06 (0.02–0.26)	0.104
Low IgA, *n*/*N* (%)	48/68 (70.6)	17/20 (85)	0.255
IgM (g/L), median (Q1–Q3)	0.4 (0.2–0.7)	0.2 (0.1–0.4)	**0.012**
Low IgM, *n*/*N* (%)	29/68 (42.6)	13/21 (61.9)	0.122
Lymphocytes (cells/μl), median (Q1–Q3)	2,828 (1,708–4,352)	2,299 (1,728–3,892)	0.610
Low lymphocyte count, *n*/*N* (%)	19/69 (27.5)	7/21 (33.3)	0.608
T cells (cells/μl), median (Q1–Q3)	1,276 (683–2,640)	908 (440–2,167)	0.196
Low T cell count, *n*/*N* (%)	41/69 (59.4)	17/21 (80.9)	0.117
CD4^+^ T cells (cells/μl), median (Q1–Q3)	346 (233–578)	307 (129–617)	0.262
Low CD4^+^ T cell count, *n*/*N* (%)	59/69 (85.5)	21/21 (100)	0.109
Inverted CD4^+^/CD8^+^ T cell ratio, *n*/*N* (%)	45/69 (65.2)	18/21 (85.7)	0.103
Low naïve CD4^+^ T cell frequency, *n*/*N* (%)	26/32 (81.3)	14/14 (100)	0.157
**Outcomes** ^c^	​	​	​
Dead, *n* (%)	39 (61.9)	16 (84.2)	0.096
Age of death (months), median (Q1–Q3)	48 (26–66)	12.5 (6.3–20.3)	**<0.001**

ENT, ear, nose, and throat.

All bold values are statistically significant p-values (p < 0.05).

a Percentages were calculated among symptomatic patients: *RFXANK* (*n* = 68) and *RFXAP* (*n* = 20).

b For immunological parameters, denominators varied according to data availability, as complete immunologic assessment was not available for all patients.

c Percentages were calculated among patients who did not undergo hematopoietic stem cell transplantation: *RFXANK* (*n* = 63) and *RFXAP* (*n* = 19).

Immunologically, patients carrying *RFXAP* c.368del showed significantly lower IgG and IgM levels (Table 3). A higher proportion of patients with low total CD4^+^ T cell counts, reversed CD4^+^/CD8^+^ T cell ratios, and low naïve CD4^+^ T cell frequencies was also observed in this group, although these differences did not reach statistical significance (Table 3). Despite the absence of a significant difference in mortality rate (61.9% versus 84.2%, P = 0.096), patients carrying the *RFXAP* c.368del mutation died at a significantly younger age than those with *RFXANK* c.338-25_338del (12.5 versus 48 mo, P < 0.001). Consistent with these findings, Kaplan–Meier survival analysis demonstrated significantly reduced survival among patients with *RFXAP* c.368del compared with those carrying *RFXANK* c.338-25_338del (median survival: 17 versus 66 mo; log-rank P < 0.001) (Fig. 4 E).

## Discussion

To the best of our knowledge, this study represents the largest cohort of patients with MHC-II deficiency reported to date and provides a comprehensive clinical, immunological, and molecular characterization of this IEI. Beyond confirming the predominance of the previously recognized founder *RFXANK* c.338-25_338del variant, our data reveal substantial enrichment of the recurrent *RFXAP* c.368del variant among Algerian patients, raising the possibility of a regional founder effect. Notably, patients carrying the latter variant displayed features suggestive of a more severe disease phenotype. In addition, the identification of four previously unreported mutations expands the mutational spectrum of the disease.

In MHC-II deficiency, clinical manifestations usually begin early, typically within the first year of life, and tend to follow a relatively consistent clinical phenotype across reported cohorts (7, 11, 12, 13). Consistent with previous reports, the hallmark manifestations in our study were recurrent respiratory infections, chronic diarrhea, and failure to thrive. Although the infectious spectrum reported in the literature includes both opportunistic intracellular pathogens and conventional bacterial and fungal pathogens (13), detailed microbiological characterization and information on the antimicrobial treatments used for individual infectious episodes were not systematically available in our cohort, representing a limitation inherent to the retrospective nature of the study. Furthermore, clinical manifestations were extracted from clinical data forms completed at admission; therefore, manifestations developing later during follow-up may not have been captured. These limitations are particularly relevant to the assessment of *Cryptosporidium*-associated hepatobiliary disease, as screening for *Cryptosporidium* infection and hepatobiliary complications was not systematic and their frequency may consequently have been underestimated.

Given the severity of this phenotype, untreated disease is frequently fatal early in life, although occasional long-term survival has been described (14). Consistent with these observations, the mortality rate among non-transplanted patients in our cohort was 66.7%. Allogeneic HSCT remains the treatment of choice, with outcomes improving substantially in recent years despite historical challenges related to infections, conditioning toxicity, graft rejection, and graft-versus-host disease (15, 16). Unfortunately, only seven patients in our cohort underwent HSCT, five of whom were transplanted in Algeria. This limited number reflects the recent implementation and restricted availability of HSCT for IEIs in Algeria. Consequently, management of most patients relied on supportive care while awaiting access to transplantation.

Classically, the immunological phenotype of MHC-II deficiency is characterized by absent or markedly reduced MHC-II expression, hypogammaglobulinemia, and CD4^+^ T cell lymphopenia with preserved total T and B cell counts. However, in our cohort, more than half of patients showed reduced total T and/or B cell counts, while some exhibited normal CD4^+^ T cell counts and/or preserved serum Ig levels, challenging the notion of a uniform “classical” immunological phenotype. This heterogeneity is consistent with current ESID diagnostic criteria, which recognize that patients may present with variable combinations of hypogammaglobulinemia, lymphopenia, reduced CD4^+^ T cell counts, and impaired antigen-specific responses (17). Nevertheless, reduced MHC-II expression remains the most consistent diagnostic hallmark. It is noteworthy that, before molecular diagnosis, two patients in our cohort were initially misdiagnosed as severe CID (SCID T⁻B⁻) due to severe lymphopenia and non-evaluation of MHC-II expression. Consistent with recommendations from other authors (11), we therefore support routine assessment of HLA-DR/MHC-II expression in all patients suspected of having CID, particularly in the context of consanguinity, to avoid missed or delayed diagnoses.

Family history also had a significant impact on the timing of diagnosis. Patients with a positive family history were diagnosed at a younger age than those without an affected relative (9 versus 17.5 mo; P = 0.001). Notably, family history facilitated both earlier recognition of the disease after symptom onset and presymptomatic diagnosis in seven subsequently born siblings through targeted neonatal screening based on HLA-DR expression. These findings highlight the importance of systematically eliciting family history and implementing targeted neonatal screening in families with a previously affected child. Such an approach may facilitate timely referral for HSCT before the development of severe infectious complications, thereby improving clinical outcomes.

This immunological heterogeneity was further supported by extended lymphocyte phenotyping. We observed a dissociation between recent thymic emigrants (RTE) and naïve CD4^+^ T cells, with reduced RTE levels in only 41% of patients compared with decreased naïve CD4^+^ T cells in 86%, suggesting that impaired thymic output alone may not fully account for CD4^+^ T cell lymphopenia. This raises the possibility that altered peripheral T cell homeostasis may contribute to depletion of the naïve CD4^+^ compartment, consistent with the recognized role of self-MHC–dependent survival signals in maintaining naïve CD4^+^ T cells (20). In the CD8 compartment, frequent reduction of naïve CD8^+^ T cells despite preserved MHC class I–dependent thymic selection may reflect chronic antigenic stimulation driving differentiation toward effector/memory subsets (21), leading to progressive depletion of the naïve pool, as observed in other IEIs (22). A further intriguing observation was the apparent preservation of SM percentages in most patients, despite defective CD4^+^ T cell help. This observation should, however, be interpreted cautiously. Most patients in our cohort were younger than 2 years, an age at which the lower reference limit for SMs is extremely low (0.1%) (23). Moreover, B cell subset analysis has limited diagnostic value during early childhood because physiological immune maturation may mask subtle pathological abnormalities (24). In this context, interpretation based on absolute counts, ideally compared with age-matched healthy controls, may have been more informative than reliance on reference intervals alone.

**Figure 5.**
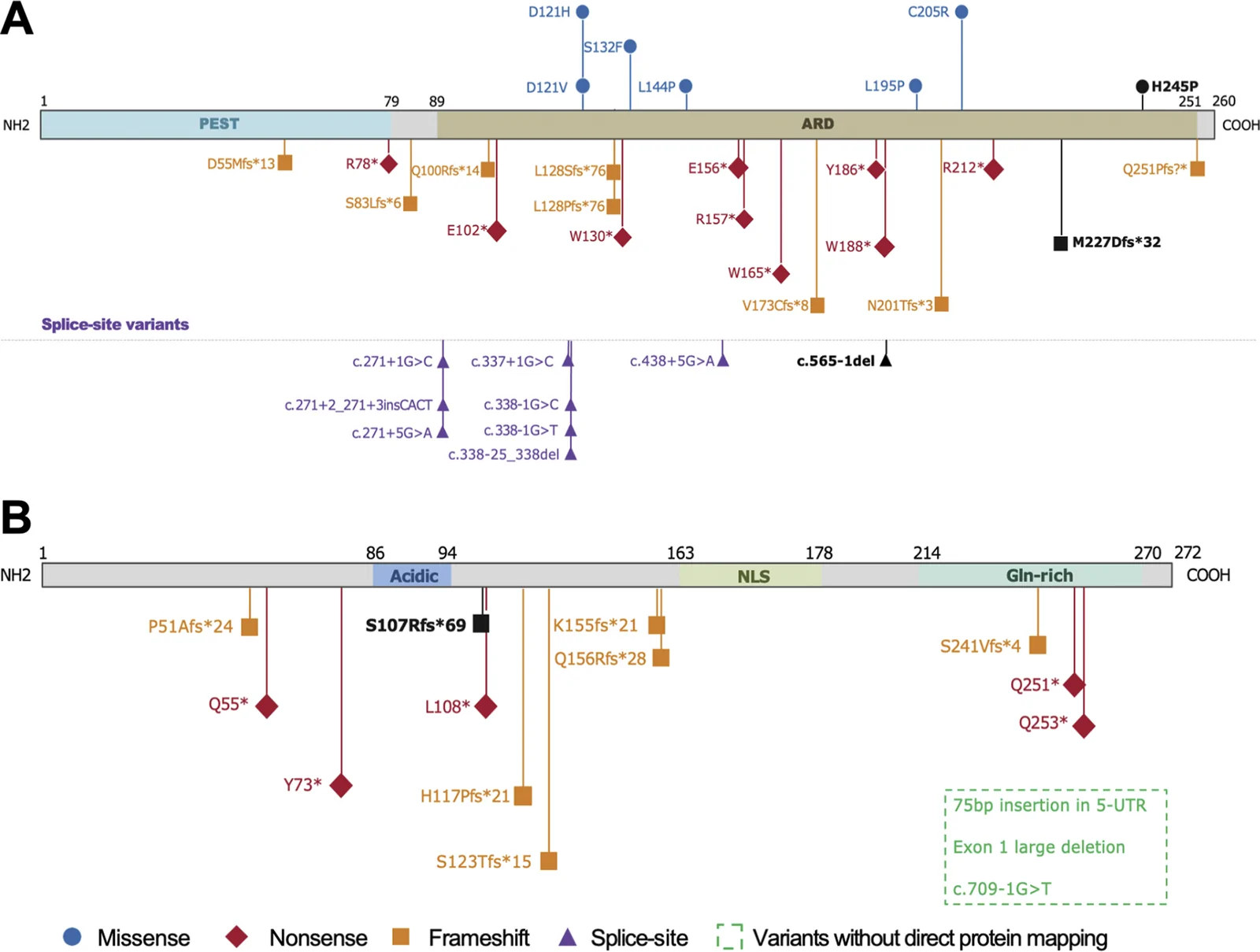
**Protein-domain mapping of *RFXANK* and *RFXAP* variants from the literature and the present study. (A)** Distribution of *RFXANK* mutations along the protein, including missense, nonsense, frameshift, and splice-site variants. Previously reported mutations are shown in color, whereas new variants identified in the present cohort are shown in black. **(B)** Distribution of *RFXAP* mutations using the same annotation. Protein domain organization adapted from Gulec Koksal et al. (13). ARD, ankyrin repeat domain; Gln, glutamine; NLS, nuclear localization signal; PEST, proline-, glutamate-, serine-, and threonine-rich region.

While absent MHC-II expression remains the cornerstone of diagnosis, identification of the underlying molecular defect is essential for definitive diagnosis and informing genetic counseling. MHC-II deficiency is caused by mutations in one of the four genes (*CIITA*, *RFXANK*, *RFX5*, and *RFXAP*) encoding transcriptional regulators of MHC-II expression (10). In our cohort, mutations were identified exclusively in *RFXANK* and *RFXAP*, whereas no variants were detected in *CIITA* or *RFX5*. The predominance of *RFXANK* mutations (77.8%) is consistent with previous reports identifying this gene as the major cause of MHC-II deficiency worldwide. To date, more than 30 pathogenic variants have been identified in this gene (Table S4 and Fig. 5 A). The most frequent variant in our cohort was the recurrent *RFXANK* c.338-25_338del mutation (formerly c.752delG26), identified in 71.7% of genetically characterized patients. Its predominance further confirms previous reports of a high frequency of this mutation in North African populations, particularly in the Maghreb (Morocco, Algeria, and Tunisia), where a founder effect was first documented by Wiszniewski et al. (5) and later supported in a larger Maghrebin cohort (7). In addition, three novel *RFXANK* variants, namely c.565-1del, c.677dup (p.Met227AspfsTer32), and c.734A>C (p.His245Pro), were identified in our cohort and classified as pathogenic or likely pathogenic according to the American College of Medical Genetics and Genomics (ACMG) criteria. Although functional studies would strengthen the interpretation of these variants, such assays were not available within our current laboratory setting. Therefore, their predicted molecular consequences could not be experimentally confirmed and should be further evaluated in future studies.

The second gene identified in our cohort was *RFXAP*, in which c.368del (p.Ser123ThrfsTer15) represented the second most frequent variant (21.2%). This mutation has previously been reported in six patients (19, 25, 26, 27) and was originally described as c.484delG. Five of these individuals were of North African origin (two Moroccan, one Algerian, and two of unspecified origin). Importantly, this variant was first reported in 1997 (19, 25). Its relatively limited representation in subsequent regional studies may partly reflect the frequent use of targeted screening for the recurrent 26-bp *RFXANK* founder deletion rather than comprehensive analysis of all MHC-II regulatory genes (8, 9, 28). The recurrence of this variant in 21 patients from 19 unrelated families in our cohort, together with its previous identification predominantly in patients of North African origin, suggests substantial enrichment among Maghrebi patients with MHC-II deficiency. Whether this enrichment reflects shared ancestry and a regional founder effect remains to be determined through haplotype-based studies. Moreover, we identified a novel mutation, c.320dup (p.Ser107ArgfsTer69), classified as pathogenic according to ACMG criteria, further expanding the mutational spectrum of *RFXAP* (Table S5 and Fig. 5 B). Its predicted molecular consequence will require confirmation through future functional studies. The predominance of two recurrent variants in our cohort suggests that a prioritized mutation-based diagnostic approach may be considered before broader genomic testing.

The presence of two well-represented mutation-defined patient groups provided a unique opportunity to explore genotype–phenotype correlations in MHC-II deficiency. Consistent with previous observations suggesting a comparatively milder disease course in patients with *RFXANK* defects (13), our data showed that carriers of the *RFXANK* c.338-25_338del mutation had later symptom onset, older age at death, and significantly longer survival than patients carrying the *RFXAP* c.368del variant. To note, survival analyses were restricted to non-transplanted patients. Moreover, the diagnostic periods substantially overlapped between the two groups, with a median year of diagnosis of 2021 (Q1–Q3: 2017–2023) in the RFXANK group and 2022 (Q1–Q3: 2020–2023) in the RFXAP group (P = 0.246). Thus, both groups were diagnosed during broadly comparable periods of supportive care. Nevertheless, given the retrospective nature of the study, residual differences in follow-up, access to supportive care, environmental exposures, and other unmeasured confounding factors cannot be excluded. Accordingly, these genotype–phenotype findings should be interpreted as exploratory.

From an immunological perspective, carriers of the *RFXAP* c.368del variant also displayed features suggestive of a more severe combined immunodeficiency phenotype. However, all patients in our cohort showed complete absence of HLA-DR expression on peripheral blood B cells and monocytes, indicating that differential HLA-DR expression is unlikely to account for the observed phenotypic variability.

An additional noteworthy finding was the exclusive occurrence of nystagmus within our cohort in patients carrying the *RFXAP* c.368del variant. Although neurological manifestations were more frequent in this group, limited and non-systematic neurological and neuroimaging assessments may have resulted in the underrecognition of neurological abnormalities in other patients, including those belonging to the other genotype groups. Moreover, neurological manifestations have previously been reported in patients with *RFXANK* variants (7, 12). Nevertheless, to our knowledge, nystagmus has not previously been reported in association with this variant (19, 25, 26, 27). Prospective studies incorporating standardized neurological and neuroradiological assessments will be required to determine the nature, frequency, and clinical significance of this phenotype.

The biological basis underlying these exploratory genotype–phenotype differences remains unknown. A recent multicenter study also reported a more severe clinical course in patients with *RFX5* deficiency than in those with *RFXANK* mutations (13), suggesting that disease severity may differ according to the affected component of the MHC-II transcriptional regulatory complex. However, functional studies are required to determine whether these differences are directly attributable to genotype.

In conclusion, this study represents the largest cohort of MHC-II deficiency reported to date and further expands current knowledge of the disease. The mutational profile identified supports a stepwise diagnostic strategy that may be particularly relevant for Algeria and neighboring Maghreb populations, prioritizing targeted screening of the *RFXANK* c.338-25_338del founder mutation, followed by the *RFXAP* c.368del variant, while patients lacking these variants should undergo broader genomic analysis using next-generation sequencing (NGS) or WES. In parallel, raising clinicians’ awareness to identify patients at risk and expanding access to genetic counseling for patients and their families will also be essential to facilitate earlier diagnosis and timely referral for HSCT. However, the successful implementation of this strategy will require the establishment of additional specialized transplantation centers with expertise in IEIs to improve access to HSCT and ultimately enhance patient outcomes in our country.

## Materials and methods

### Study population

107 patients with MHC-II deficiency, initially defined by absent or markedly reduced HLA-DR expression on B cells and monocytes, were enrolled in this retrospective study. Patients from different provinces of Algeria (Fig. 1 and Table S1) were initially evaluated by pediatricians at multiple hospitals nationwide and were subsequently referred to the Department of Medical Immunology at Beni-Messous University Hospital Center for immunological assessment.

Data from eligible patients were collected using a data collection form implemented in an encrypted Microsoft Excel spreadsheet to record demographic information, medical history, laboratory, and molecular findings.

Written informed consent was obtained from the parents or legal guardians of all participants. The study was conducted in accordance with the principles of the Declaration of Helsinki and was approved by the Ethics Committee of Beni-Messous University Hospital Center, Algiers, Algeria (Approval No.: 17/CE/2026).

### Immunological analysis

Initial investigations included a complete blood count performed on an automated hematological analyzer, measurement of total serum Ig’s (IgM, IgG, and IgA) by nephelometry (BN ProSpec, Siemens), and analysis of lymphocyte subsets (T cells [CD3^+^, CD4^+^, and CD8^+^], B cells [CD19^+^], and NK cells [CD56^+^, CD16^+^]) by flow cytometry (BD FACS Lyric [Becton Dickinson]).

A low absolute CD4^+^ T cell count was the main criterion for flow cytometric evaluation of HLA-DR expression on B cells and monocytes. Nevertheless, HLA-DR expression was also assessed in patients with a strong clinical suspicion of MHC-II deficiency, even if the CD4^+^ T cell count was normal, as well as in asymptomatic individuals screened during the neonatal period because of a familial history of the disease.

The flow cytometry analysis was completed by extended phenotyping of peripheral T and B cell compartments. T cell subsets were defined as follows: RTE CD4^+^ T cells (CD45RA^+^, CD31^+^), naïve T cells (CD45RA^+^CD27^+^), central memory T cells (CD45RA^−^CD27^+^), EM (CD45RA^−^CD27^−^), and TEMRA (CD45RA^+^CD27^−^). B cell subsets included: SM (SM; CD27^+^, IgD^−^) and nSM subsets (CD27^+^, IgD^+^), transitional B cells (CD24^++^, CD38^++^), plasmablasts (CD24^−^, CD38^++^), and CD21^–/low^ B cells. Detailed antibody panels are provided in Table S2, and the gating strategies are shown in Figs. S1 and S2.

Serum IgG, IgA, and IgM concentrations were interpreted using age-adjusted, laboratory-specific reference intervals provided by Mayo Clinic Laboratories and reproduced by DiGiacomo and Barmettler (29, 30). Lymphocyte subset counts and percentages were interpreted against age-specific pediatric reference ranges (23, 31).

### Genetic analysis

Genomic DNA was extracted from whole blood using either the salting-out method, a silica column–based extraction technique, or a magnetic bead–based method, depending on reagent and equipment availability. Overall, genetic material was available for 99 of the 107 patients (92.5%).

Before the establishment of the molecular assay platform in our department in 2025, international academic collaboration enabled the molecular diagnosis of 68 patients using WES. Exome capture was performed using the SureSelect Human All Exon V7 kit (Agilent Technologies) or the KAPA HyperPrep Kit (Roche Sequencing Solutions), followed by 150-bp paired-end sequencing on a NovaSeq 6000 platform (Illumina). All identified variants were subsequently confirmed by bidirectional targeted Sanger sequencing using an Applied Biosystems 3730xl DNA Analyzer (Thermo Fisher Scientific).

After the platform became operational, the remaining 31 patients were analyzed locally. In accordance with MENA regional recommendations (32), targeted Sanger sequencing was initially performed to screen for the North African common founder mutation c.338-25_338del in the *RFXANK* gene. PCR products covering the genomic region Chr19:19,197,106-19,197,612 (GRCh38), encompassing exons 5 and 6 and the intervening intron, were directly sequenced using the SeqStudio Genetic Analyzer (Thermo Fisher Scientific), and sequences were analyzed using SeqScape software. Patients negative for this variant were subsequently analyzed by NGS using a custom-designed panel including the four genes implicated in MHC-II deficiency. Library and template preparation were performed on the Ion Chef System (Thermo Fisher Scientific), followed by sequencing on the Ion S5 System (Thermo Fisher Scientific) according to the manufacturer’s instructions.

The reference transcript sequences NM_003721.4 (*RFXANK*) and NM_000538.4 (*RFXAP*) were used for sequence alignment and variant nomenclature according to the Human Genome Variation Society (HGVS) recommendations. Any suspected mutations found were compared with the online databases and the literature to determine if they had been previously reported (as of August 2026). Novel variants were analyzed using Mutalyzer for HGVS validation and annotation of variant consequences, whereas pathogenicity was assessed using in silico prediction tools (PolyPhen-2, SIFT, etc.). Variant classification was subsequently performed according to the ACMG guidelines (33).

### Statistical analysis

IBM SPSS Statistics (version 20.0) was used for statistical analysis. Categorical variables were expressed as absolute numbers (*n*) or relative frequencies (%), and significance was assessed by χ^2^ test or Fisher’s exact test, as appropriate. Continuous variables were expressed as medians and interquartile ranges (Q1–Q3). As these variables were non-normally distributed, differences between two independent groups were assessed using the Mann–Whitney U test. Survival analyses were restricted to patients known not to have undergone HSCT. Survival time was measured from birth to death or, for patients confirmed to be alive, to the administrative end of follow-up in July 2026. The vital status of all surviving patients was verified at that time, and they were right-censored at the age reached in July 2026. Patients with unknown transplantation or vital status, as well as those lacking time-to-event data, were excluded from the survival analyses. Survival probabilities were estimated using the Kaplan–Meier method and compared between groups using the log-rank test. P values <0.05 were considered statistically significant for all analyses.

### Online supplemental material

Figs. S1 and S2 show the flow cytometry gating strategies used for lymphocyte immunophenotyping and HLA-DR expression. Fig. S3 shows conservation of the *RFXANK* p.His245 residue across vertebrates and its position within the RFXANK protein. Fig. S4 shows HLA-DR expression in patients carrying the novel variants. Table S1 presents the geographic distribution of patients with MHC-II deficiency across Algerian provinces. Table S2 details the flow cytometry panels used for lymphocyte immunophenotyping. Table S3 summarizes the characteristics and posttransplant immunological findings of patients who underwent HSCT. Tables S4 and S5 summarize previously reported *RFXANK* and *RFXAP* variants, respectively.

## Supplementary Material

Table S1 shows geographic distribution of patients with MHC-II deficiency across Algerian provinces. Data were available for 103 patients.

Table S2 shows flow cytometry panels used in lymphocyte immunophenotyping.

Table S3 shows HSCT characteristics and outcomes.

Table S4 shows published *RFXANK* variants reported in MHC-II deficiency.

Table S5 shows published *RFXAP* variants reported in MHC-II deficiency.

## Data Availability

The clinical, biological, and genetic data supporting the findings of this study are not publicly available due to patient privacy and ethical restrictions. Requests for access to de-identified data should be directed to the corresponding author, Prof. Reda Djidjik (r.djidjik@univ-alger.dz). Access may be granted upon reasonable request, subject to applicable institutional and ethical requirements and the scope of participants’ informed consent. The novel *RFXANK* and *RFXAP* variants identified in this study have been submitted to ClinVar. Their accession numbers are SCV007680856, SCV007680857, SCV007680858, and SCV007680859.
