# Supplementary material for: MHC-II deficiency in a large Algerian cohort: Clinical, immunological, and genetic characterization
Source: J Hum Immun. 2026 Oct 30;3(1):e20260162. doi: 10.70962/jhi.20260162 (PMC13721797; doi:10.70962/jhi.20260162)
Supplement: Supplementary file 1 [file jhi_20260162_tables1.docx]

**Table S1.** Geographic distribution of patients with MHC class-II deficiency across Algerian provinces. Data were available for 103 patients.

| **Province** | **N** | **Province** | **N** | **Province** | **N** | **Province** | **N** |
| --- | --- | --- | --- | --- | --- | --- | --- |
| Algiers | 13 | Tizi Ouzou | 4 | Constantine | 2 | Bouira | 1 |
| Ain Defla | 10 | Batna | 3 | Relizane | 2 | Jijel | 1 |
| Tlemcen | 9 | Chlef | 3 | Saida | 2 | M’sila | 1 |
| Oran | 8 | Djelfa | 3 | Tiaret | 2 | Ouargla | 1 |
| Boumerdes | 6 | Mascara | 3 | Ain Timouchent | 1 | Souk Ahras | 1 |
| Tissemsilt | 6 | Medea | 3 | Annaba | 1 | Timimoun | 1 |
| Blida | 4 | Setif | 3 | Bordj Bouarreridj | 1 | Touggourt | 1 |
| Ghardaia | 4 | Bejaia | 2 | Biskra | 1 |  |  |
