# Supplementary material for: MHC-II deficiency in a large Algerian cohort: Clinical, immunological, and genetic characterization
Source: J Hum Immun. 2026 Oct 30;3(1):e20260162. doi: 10.70962/jhi.20260162 (PMC13721797; doi:10.70962/jhi.20260162)
Supplement: Supplementary file 2 [file jhi_20260162_tables2.docx]

**Table S2.** Flow cytometry panels used in lymphocyte immunophenotyping

| Antigen/Marker | Fluorochrome | Clone | Manufacturer |
| --- | --- | --- | --- |
| **T-B-NK Panel:** general lymphocyte overview | | | |
| CD45 | PerCP-Cy5.5 | 2D1 | BD Biosciences |
| CD3 | FITC | SK7 | BD Biosciences |
| CD4 | PE-Cy7 | SK3 | BD Biosciences |
| CD8 | APC-Cy7 | SK1 | BD Biosciences |
| CD19 | APC | SJ25C1 | BD Biosciences |
| CD16/56 | PE | B73.1/NCAM16.2 | BD Biosciences |
| **HLA DR expression** | | | |
| CD19 | PerCP-Cy5.5 | SJ25C1 | BD Biosciences |
| HLA-DR | FITC | L243 | BD Biosciences |
| **Extended T panel:** naive and memory T-cell subsets | | | |
| CD3 | PerCP-Cy5.5 | SK7 | BD Biosciences |
| CD4 | APC | SK3 | BD Biosciences |
| CD8 | PE-Cy7 | SK1 | BD Biosciences |
| CD27 | V500 | M-T271 | BD Biosciences |
| CD45RA | FITC | L48 | BD Biosciences |
| CD45RO | PE | UCHL1 | BD Biosciences |
| CD31 | APC-Cy7 | WM59 | BD Pharmingen |
| **Extended B panel:** B-cell subsets | | | |
| CD19 | V450 | SJ25C1 | BD Biosciences |
| IgD | APC | IA6-2 | BD Pharmingen |
| CD27 | PE | L128 | BD Biosciences |
| CD21 | FITC | B-ly4 | BD Pharmingen |
| CD24 | PE-Cy7 | ML5 | BD Pharmingen |
| CD38 | PerCP-Cy5.5 | HIT2 | BD Pharmingen |

**APC**: allophycocyanine; **CD**: cluster of differentiation; **Cy**: cyanine; **FITC**: Fluorescein isothiocyanate; **PE**: phycoerythrin; **PerCp**: peridinin-Chlorophyll-protein; **V**: violet.
