# Supplementary material for: MHC-II deficiency in a large Algerian cohort: Clinical, immunological, and genetic characterization
Source: J Hum Immun. 2026 Oct 30;3(1):e20260162. doi: 10.70962/jhi.20260162 (PMC13721797; doi:10.70962/jhi.20260162)
Supplement: Supplementary file 3 [file jhi_20260162_tables3.docx]

**Table S3.** HSCT characteristics and outcomes.

| **Patients** | **1** | **2** | **3** | **4** | **5*** | **6** | **7** |
| --- | --- | --- | --- | --- | --- | --- | --- |
| **Age at diagnosis** | 5y 2m | 4y 9m | 1y 3m | 3y | 6y | 2d (screening) | 1m 15d (screening) |
| **Age at HSCT** | 6y 5m | 7y 9m | 6y 3m | 4y 6m | 10y | NA | NA |
| **Country of transplantation** | Algeria | Algeria | Algeria | Algeria | Algeria | Türkiye | Türkiye |
| **Stem cell source** | BM | BM | BM | BM | BM | BM | BM |
| **HLA match** | MSD | MSD | MSD | MSD | MSD | NA | NA |
| **Conditioning regimen and intensity** | Flu + Bu  Reduced intensity | Flu + Bu  Reduced intensity | Flu + Bu  Reduced intensity | Flu + Bu  Reduced intensity | Flu + Bu  Reduced intensity | NA | NA |
| **GvHD prophylaxis** | Cs + MTX | Cs + MTX | Cs + MTX | Cs + MTX | Cs + MTX | NA | NA |
| **GvHD** | No | Acute GvHD (grade II skin) | Chronic GvHD (bronchiolitis obliterans) | No | No | NA | NA |
| **Major post-transplant complications** | Hypertension Bronchopneumonia | No other reported complications | Pulmonary infection Lymphocytic meningitis | Dacryocystitis | Pneumonia  Dilated cardiomyopathy | NA | NA |
| **Last available donor chimerism results** | 100% at 6 months post-HSCT | 100% at 9 months post-HSCT | 99% on day 99 post-HSCT | 96% at 6 months post-HSCT | 88% on day 30 post-HSCT | NA | NA |
| **Time since HSCT at immunological evaluation** | 2y 5m | 2y 6m | 2y 8m | 8m | NA | NA | NA |
| **IgG (g/L)** | 16  [4.62-16.8] | 10.9  [4.62-16.8] | NA | NA | NA | NA | NA |
| **IgA (g/L)** | 1.6  [0.34-2.74] | 1.5  [0.34-2.74] | NA | NA | NA | NA | NA |
| **IgM (g/L)** | 1.1  [0.38-2.51] | 1.6  [0.38-2.51] | NA | NA | NA | NA | NA |
| **HLA-DR expression (%)** | 97% | 99% | 98% | 100% | NA | NA | NA |
| **Lymphocytes (cells/µL)** | 3411  [1200-2700] | 3077  [1200-2700] | 2280  [1200-2700] | NA | NA | NA | NA |
| **T cells (cells/µL)** | 2429  [1325-2276] | 2052  [1325-2276] | 1809  [1325-2276] | NA | NA | NA | NA |
| **LTCD4+ (cells/µL)** | 843  [531-1110] | 1044  [531-1110] | **339**  [531-1110] | NA | NA | NA | NA |
| **LTCD8+ (cells/µL)** | 1405  [480-1112] | 880  [480-1112] | 1277  [480-1112] | NA | NA | NA | NA |
| **CD4+/CD8+ ratio** | **0.6**  [0.81-1.66] | 1.19  [0.81-1.66] | **0.27**  [0.81-1.66] | NA | NA | NA | NA |
| **B cells (cells/µL)** | 429  [216-536] | 748  [216-536] | NA | NA | NA | NA | NA |
| **NK cells (cells/µL)** | 599  [246-792] | 308  [246-792] | NA | NA | NA | NA | NA |
| **RTE (%)** | **14.3**  [19.4-60.9] | 23.9  [19.4-60.9] | 33.2  [19.4-60.9] | NA | NA | NA | NA |
| **CD4_naifs (%)** | **16.5**  [39.7-69.6] | **27.9**  [39.7-69.6] | **18.9**  [39.7-69.6] | NA | NA | NA | NA |
| **CD4_CM (%)** | 52.3  [24.2-52.7] | 52.8  [24.2-52.7] | 55.2  [24.2-52.7] | NA | NA | NA | NA |
| **CD4_EM (%)** | 31.1  [3.4-11.2] | 19.2  [3.4-11.2] | 25.7  [3.4-11.2] | NA | NA | NA | NA |
| **CD4_TEMRA (%)** | 0.1  [0.1-1.29] | 0.1  [0.1-1.29] | 0.2  [0.1-1.29] | NA | NA | NA | NA |
| **CD8_naifs (%)** | **17.1**  [41.4-73] | 70.7  [41.4-73] | **13.4**  [41.4-73] | NA | NA | NA | NA |
| **CD8_CM (%)** | 18.2  [13.2-37.9] | 24.8  [13.2-37.9] | 16  [13.2-37.9] | NA | NA | NA | NA |
| **CD8_EM (%)** | 29.2  [1.53-15.4] | 3.2  [1.53-15.4] | 52  [1.53-15.4] | NA | NA | NA | NA |
| **CD8_TEMRA (%)** | 35.5  [2.01-21.7] | **1.3**  [2.01-21.7] | 18.6  [2.01-21.7] | NA | NA | NA | NA |
| **B_nSM (%)** | **5.5**  [7.5-12.4] | **2.6**  [7.5-12.4] | NA | NA | NA | NA | NA |
| **B_SM (%)** | 15.9  [5.2-12.1] | 5.6  [5.2-12.1] | NA | NA | NA | NA | NA |
| **B_Trans (%)** | 11.3  [2.5-9.07] | 13.4  [2.5-9.07] | NA | NA | NA | NA | NA |
| **B_Plasm (%)** | 15.4  [0.7-5.67] | 1.7  [0.7-5.67] | NA | NA | NA | NA | NA |
| **B_CD21^low^ (%)** | 15.2  [0.9-3.1] | 1.8  [0.9-3.1] | NA | NA | NA | NA | NA |
| **Outcome (at last clinical follow-up)** | Alive (2 years and 5 months post-HSCT) | Alive (2 years and 6 months post-HSCT) | Alive (4 years and 3 months post-HSCT) | Alive (4 years and 2 months post-HSCT) | Dead (43 days post-HSCT due to Pneumonia) | Alive | Dead |

* Follow-up for patient “5” ended 43 days post-transplantation due to death from pneumonia.

Reference intervals are shown in square brackets. Bold values indicate results below the normal reference range.

BM, bone marrow; Bu, busulfan; CM, central memory; Cs, cyclosporine; EM, effector memory; Flu, fludarabine; GvHD, graft versus host disease; HSCT, hematopoietic stem–cell transplantation; MSD, matched sibling donor; MTX, methotrexate; NK, natural killer; nSM, non-switched memory; Plasm, plasmablasts; RTE, recent thymic emigrant; TEMRA, terminally differentiated effector memory re-expressing CD45RA; Trans, transitional; SM, switched memory.
