# Supplementary material for: MHC-II deficiency in a large Algerian cohort: Clinical, immunological, and genetic characterization
Source: J Hum Immun. 2026 Oct 30;3(1):e20260162. doi: 10.70962/jhi.20260162 (PMC13721797; doi:10.70962/jhi.20260162)
Supplement: Supplementary file 4 [file jhi_20260162_tables4.docx]

**Table S4.** Published *RFXANK* variants reported in MHC class II deficiency

| **Mutation** | **Case number** | **Ethnicity** | **Author** | **Year of publication** | **Reference** |
| --- | --- | --- | --- | --- | --- |
| c.338-25_338del  (*Formerly I5E6-25_I5E6+1*  *752delG-25*  *752delG26*) | 3  2  17  1  10  35  2  9  25  34 | NR  NR  Algerian (13)  Tunisian (3)  Moroccan (1)  NR  Moroccan  Algerian (25)  Moroccan (4)  Tunisian (6)  Tunisian  Algerian  Tunisian  Moroccan | Masternak et al  Nagarajan et al.  Wiszniewski et al  Nagarajan et al  Naamane et al  Ouederni et al  Guirat-Dhouib et al  Djidjik et al  Ben-Mustapha et al  Kattra et al | 1998  1999  2000  2000  2010  2011  2012  2012  2013  2026 | [1]  [2]  [3]  [4]  [5]  [6]  [7]  [8]  [9]  [10] |
| c.584 T>C  p.Leu195Pro | 1 | NR | Nagarajan et al | 2000 | [4] |
| c.304G>T  p.Glu102Ter  *(Formerly G721T*  *p.Glu103X or p. Glu101X)* | 1  1  1 | NR  Turkish  Turkish | Nagarajan et al  Wiszniewski et al  Gulec Koksal et al | 2000  2000  2024 | [4]  [3]  [11] |
| *Formerly C883T*  *p.Glu156Ter* | 1 | Italian | Wiszniewski et al | 2000 | [3] |
| Compound heterozygous  *Formerly 797delT/856-1G>T* | 1 | French/Spanish | Wiszniewski et al | 2000 | [3] |
| c.271+1G>C  (*Formerly c. IVS4+1G>C*) | 1  2 | North Africain  Saudi Arabian | Lennon-Duménil et al  Alharby et al | 2001  2021 | [12]  [13] |
| c.362A>T  p.Asp121Val | 1  4  1  1  1 | Saudi Arabian  Kuwaiti  Saudi Arabian  Iranian  Iranian | Wiszniewski et al  Al-Herz et al  Abu-Arja et al  Shah et al  Ramanathan et al | 2003  2012  2014  2017  2022 | [14]  [15]  [16]  [17]  [18] |
| c.634C>T  p.Arg212Ter | 1  1  2 | Turkish  Iranian  Turkish | Wiszniewski et al  Musavi Khorshidi et al  Gulec Koksal et al | 2003  2023  2024 | [14]  [19]  [11] |
| c.271+5G>A  (*Formerly c.IVS4+5G>A*) | 1 | Jewish-Egyptian | Prod’hommeet al | 2003 | [20] |
| c.563G>A  p.Trp188Ter  (*Formerly c.564G>A*) | 2  1  1 | Kuwaiti  Iranian  Iranian | Al-Herz et al  Farrokhi et al  Musavi Khorshidi et al | 2012  2018  2023 | [15]  [21]  [19] |
| c.271+2_271+3insCACT  (*Formerly c.IVS4+1insTCAC*) | 4 | Kuwaiti | Al-Herz et al | 2012 | [15] |
| c.469C>T  p.Arg157Ter | 1 | Mexican | Clarridge et al | 2015 | [22] |
| c.438+5G>A | 1  2 | Iranian  Iranian | Sheikhbahaei et al  Musavi Khorshidi et al | 2016  2023 | [23]  [19] |
| c.431T>C  p.Leu144Pro | 2 | Egyptian | El Hawary et al | 2018 | [24] |
| c.247_250del  p.Ser83LeufsTer6 | 1 | Egyptian | El Hawary et al | 2018 | [24] |
| c.600del  p.Asn201ThrfsTer3 | 1 | Egyptian | El Hawary et al | 2018 | [24] |
| c.495G>A  p.Trp165Ter | 1 | Iranian | Abolnezhadian et al | 2018 | [25] |
| c. 337 + 1G>C | 1 | Chinese | Cai et al | 2020 | [26] |

| **Mutation** | **Case number** | **Ethnicity** | **Author** | **Year of publication** | **Reference** |
| --- | --- | --- | --- | --- | --- |
| c.162del  p.Asp55MetfsTer13 | 1  6  1 | Iranian  Iranian  Turkish | Abolnezhadian et al  Musavi Khorshidi et al  Gulec Koksal et al | 2020  2023  2024 | [27]  [19]  [11] |
| c.232C > T  p.Arg78Ter | 1 | Afghan | Damoiseaux et al | 2022 | [28] |
| c.338-1G>C | 1 | Iranian | Musavi Khorshidi et al | 2023 | [19] |
| c.361G>C  p.Asp121His | 1 | Iranian | Musavi Khorshidi et al | 2023 | [19] |
| c.390G>A  p.Trp130Ter | 1 | Iranian | Musavi Khorshidi et al | 2023 | [19] |
| Compound heterozygous  c.304G>T/c.634C>T  p.Glu102Ter/p.Arg212 Ter | 1 | Turkish | Ünsal et al | 2023 | [29] |
| c.558T>A  p.Tyr186Ter | 1 | Saudi Arabian | Alroqi et al | 2024 | [30] |
| c.516dup  p.Val173CysfsTer8 | 1 | Chinese | Li et al | 2024 | [31] |
| c.338-1G>T | 1 | Turkish | Gulec Koksal et al | 2024 | [11] |
| c.299_300del  p.Gln100ArgfsTer14 | 1 | Turkish | Gulec Koksal et al | 2024 | [11] |
| c.613T>C  p.Cys205Arg | 1 | Turkish | Gulec Koksal et al | 2024 | [11] |
| c.382del  p.Leu128SerfsTer76 | 1 | Indian | Aswanth et al | 2025 | [32] |
| c.383del  p.Leu128ProfsTer76 | 1 | Mexican | Nunez-Nunez et al | 2026 | [33] |
| c.395C>T  p.Ser132Phe | 1 | Turkish | Haskologlu et al | 2026 | [34] |
| c.752_767del  p.Gln251Profs?* | 1 | Turkish | Haskologlu et al | 2026 | [34] |

**NR:** not reported.

**References**

1.	Masternak K, Barras E, Zufferey M, Conrad B, Corthals G, Aebersold R, et al. A gene encoding a novel RFX-associated transactivator is mutated in the majority of MHC class II deficiency patients. Nat Genet. nov 1998;20(3):273‑7. doi:10.1038/3081

2.	Nagarajan UM, Louis-Plence P, DeSandro A, Nilsen R, Bushey A, Boss JM. RFX-B is the gene responsible for the most common cause of the bare lymphocyte syndrome, an MHC class II immunodeficiency. Immunity. févr 1999;10(2):153‑62. doi:10.1016/s1074-7613(00)80016-3 PubMed PMID: 10072068.

3.	Wiszniewski W, Fondaneche MC, Lambert N, Masternak K, Picard C, Notarangelo L, et al. Founder effect for a 26-bp deletion in the RFXANK gene in North African major histocompatibility complex class II-deficient patients belonging to complementation group B. Immunogenetics. 1 avr 2000;51(4):261‑7. doi:10.1007/s002510050619

4.	Nagarajan UM, Peijnenburg A, Gobin SJ, Boss JM, van den elsen PJ. Novel mutations within the RFX-B gene and partial rescue of MHC and related genes through exogenous class II transactivator in RFX-B-deficient cells. J Immunol. 1 avr 2000;164(7):3666‑74. doi:10.4049/jimmunol.164.7.3666 PubMed PMID: 10725724.

5.	Naamane H, El Maataoui O, Ailal F, Barakat A, Bennani S, Najib J, et al. The 752delG26 mutation in the RFXANK gene associated with major histocompatibility complex class II deficiency: evidence for a founder effect in the Moroccan population. Eur J Pediatr. 1 sept 2010;169(9):1069‑74. doi:10.1007/s00431-010-1179-6

6.	Ouederni M, Vincent QB, Frange P, Touzot F, Scerra S, Bejaoui M, et al. Major histocompatibility complex class II expression deficiency caused by a RFXANK founder mutation: a survey of 35 patients. Blood. 10 nov 2011;118(19):5108‑18. doi:10.1182/blood-2011-05-352716 PubMed PMID: 21908431.

7.	Guirat-Dhouib N, Baccar Y, Mustapha IB, ouederni M, Chouaibi S, El Fekih N, et al. Oral HPV infection and MHC class II deficiency (A study of two cases with atypical outcome). Clin Mol Allergy CMA. 23 avr 2012;10:6. doi:10.1186/1476-7961-10-6 PubMed PMID: 22524894; PubMed Central PMCID: PMC3349517.

8.	Djidjik R, Messaoudani N, Tahiat A, Meddour Y, Chaib S, Atek A, et al. Clinical, immunological and genetic features in eleven Algerian patients with major histocompatibility complex class II expression deficiency. Allergy Asthma Clin Immunol. 3 août 2012;8(1):14. doi:10.1186/1710-1492-8-14

9.	Ben-Mustapha I, Ben-Farhat K, Guirat-Dhouib N, Dhemaied E, Larguèche B, Ben-Ali M, et al. Clinical, Immunological and Genetic Findings of a Large Tunisian Series of Major Histocompatibility Complex Class II Deficiency Patients. J Clin Immunol. 1 mai 2013;33(4):865‑70. doi:10.1007/s10875-013-9863-8

10.	Kattra AB, Ailal F, Benhsaien I, Rada N, Admou B, Hazime R, et al. Major Histocompatibility Complex Class II Deficiency in Morocco: A 26-Year Retrospective Cohort Study. Front Immunol. 20 juill 2026;17. doi:10.3389/fimmu.2026.1831840

11.	Gulec Koksal Z, Bilgic Eltan S, Topyildiz E, Sezer A, Keles S, Celebi Celik F, et al. MHC Class II Deficiency: Clinical, Immunological, and Genetic Insights in a Large Multicenter Cohort. J Allergy Clin Immunol Pract. 1 sept 2024;12(9):2490-2502.e6. doi:10.1016/j.jaip.2024.06.046

12.	Lennon-Duménil AM, Barbouche MR, Vedrenne J, Prod’Homme T, Béjaoui M, Ghariani S, et al. Uncoordinated HLA-D gene expression in a RFXANK-defective patient with MHC class II deficiency. J Immunol. 1 mai 2001;166(9):5681‑7. doi:10.4049/jimmunol.166.9.5681 PubMed PMID: 11313409.

13.	Alharby E, Obaid M, Elamin MAO, Almuntashri M, Bakhsh I, Samman M, et al. Progressive Ataxia and Neurologic Regression in RFXANK-Associated Bare Lymphocyte Syndrome. Neurol Genet. 9 avr 2021;7(3):e586. doi:10.1212/NXG.0000000000000586 PubMed PMID: 33855173; PubMed Central PMCID: PMC8042776.

14.	Wiszniewski W, Fondaneche MC, Louise-Plence P, Prochnicka-Chalufour A, Selz F, Picard C, et al. Novel mutations in the RFXANK gene: RFX complex containing in-vitro-generated RFXANK mutant binds the promoter without transactivating MHC II. Immunogenetics. 1 févr 2003;54(11):747‑55. doi:10.1007/s00251-002-0521-1

15.	Al-Herz W, Alsmadi O, Melhem M, Recher M, Frugoni F, Notarangelo LD. Major Histocompatibility Complex Class II Deficiency in Kuwait: Clinical Manifestations, Immunological Findings and Molecular Profile. J Clin Immunol. 1 avr 2013;33(3):513‑9. doi:10.1007/s10875-012-9831-8

16.	Abu-Arja RF, Gonzalez BE, Jacobs MR, Cabral L, Egler R, Auletta J, et al. Disseminated Bacillus Calmette-Guérin (BCG) infection following allogeneic hematopoietic stem cell transplant in a patient with Bare Lymphocyte Syndrome type II. Transpl Infect Dis Off J Transplant Soc. oct 2014;16(5):830‑7. doi:10.1111/tid.12263 PubMed PMID: 24995715; PubMed Central PMCID: PMC4876699.

17.	Shah RM, Waugh S, Ng KF, Gennery AR, Slatter M, Cant AJ. Enterovirus-Related Immune Reconstitution Inflammatory Syndrome (IRIS) Following Haploidentical Stem Cell Transplantation in an MHC Class II-Deficient Child. J Clin Immunol. 1 juill 2017;37(5):419‑21. doi:10.1007/s10875-017-0402-x

18.	Ramanathan S, Veramendi-Espinoza L, Shillitoe B, Flinn A, Owens S, Williams E, et al. Haploidentical CD3+ TCR αβ/CD19+–depleted HSCT for MHC class II deficiency and persistent SARS-CoV-2 pneumonitis. J Allergy Clin Immunol Glob. 4 oct 2022;2(1):101‑4. doi:10.1016/j.jacig.2022.08.006 PubMed PMID: 36210925; PubMed Central PMCID: PMC9531933.

19.	Mousavi Khorshidi MS, Seeleuthner Y, Chavoshzadeh Z, Behfar M, Hamidieh AA, Alimadadi H, et al. Clinical, Immunological, and Genetic Findings in Iranian Patients with MHC-II Deficiency: Confirmation of c.162delG RFXANK Founder Mutation in the Iranian Population. J Clin Immunol. 1 nov 2023;43(8):1941‑52. doi:10.1007/s10875-023-01562-z

20.	Prod’homme T, Dekel B, Barbieri G, Lisowska-Grospierre B, Katz R, Charron D, et al. Splicing defect in RFXANK results in a moderate combined immunodeficiency and long-duration clinical course. Immunogenetics. 1 nov 2003;55(8):530‑9. doi:10.1007/s00251-003-0609-2

21.	Farrokhi S, Shabani M, Aryan Z, Zoghi S, Krolo A, Boztug K, et al. MHC class II deficiency: Report of a novel mutation and special review. Allergol Immunopathol (Madr). 1 mai 2018;46(3):263‑75. doi:10.1016/j.aller.2017.04.006

22.	Clarridge K, Leitenberg D, Loechelt B, Picard C, Keller M. Major Histocompatibility Complex Class II Deficiency due to a Novel Mutation in RFXANK in a Child of Mexican Descent. J Clin Immunol. 1 janv 2016;36(1):4‑5. doi:10.1007/s10875-015-0219-4

23.	Sheikhbahaei S, Sherkat R, Roos D, Yaran M, Najafi S, Emami A. Gene mutations responsible for primary immunodeficiency disorders: A report from the first primary immunodeficiency biobank in Iran. Allergy Asthma Clin Immunol Off J Can Soc Allergy Clin Immunol. 2 déc 2016;12:62. doi:10.1186/s13223-016-0166-5 PubMed PMID: 27980538; PubMed Central PMCID: PMC5133745.

24.	El Hawary RE, Mauracher AA, Meshaal SS, Eldash A, Abd Elaziz DS, Alkady R, et al. MHC-II Deficiency Among Egyptians: Novel Mutations and Unique Phenotypes. J Allergy Clin Immunol Pract. mars 2019;7(3):856‑63. doi:10.1016/j.jaip.2018.07.046 PubMed PMID: 30170160.

25.	Abolnezhadian F, Saeedi-Boroujeni A, Iranparast S. MHC Class II Deficiency with Normal CD4+ T Cell Counts: A Case Report. Iran J Allergy Asthma Immunol. 4 déc 2018;17(6):594‑600. PubMed PMID: 30644704.

26.	Cai YQ, Zhang H, Wang XZ, Xu C, Chao YQ, Shu Y, et al. A Novel RFXANK Mutation in a Chinese Child With MHC II Deficiency: Case Report and Literature Review. Open Forum Infect Dis. 24 juill 2020;7(8):ofaa314. doi:10.1093/ofid/ofaa314 PubMed PMID: 32875002; PubMed Central PMCID: PMC7452370.

27.	Abolnezhadian F, Dehghani R, Dehnavi S, Khodadadi A, Shohan M. A novel mutation in RFXANK gene and low B cell count in a patient with MHC class II deficiency: a case report. Immunol Res. 1 août 2020;68(4):225‑31. doi:10.1007/s12026-020-09141-9

28.	Damoiseaux M, Damoiseaux J, Pico-Knijnenburg I, van der Burg M, Bredius R, van Well G. Lessons learned from the diagnostic work-up of a patient with the bare lymphocyte syndrome type II. Clin Immunol. 1 févr 2022;235:108932. doi:10.1016/j.clim.2022.108932

29.	Ünsal H, Caka C, Bildik HN, Esenboğa S, Kupesiz A, Kuşkonmaz B, et al. A large single-center cohort of bare lymphocyte syndrome: Immunological and genetic features in Turkey. Scand J Immunol. 2024;99(1):e13335. doi:10.1111/sji.13335

30.	Alroqi FJ, Alhezam MA, Almojali AI, Barhoumi T, Althubaiti N, Alharbi Y, et al. Novel Presentation of Major Histocompatibility Complex Class II Deficiency with Hemophagocytic Lymphohistiocytosis. J Clin Immunol. 1 mars 2024;44(3):73. doi:10.1007/s10875-024-01674-0

31.	Li X, Lu B, Luo X. New mutations and new phenotypes: a case of Major Histocompatibility Complex Class II Deficiency. Immunol Res. déc 2024;72(6):1268‑76. doi:10.1007/s12026-024-09526-0 PubMed PMID: 39136810; PubMed Central PMCID: PMC11618159.

32.	KS. A, Satapathy D, Arun Babu T. Unmasking MHC Class II Deficiency: A Novel Mutation in a Child with Pediatric ARDS due to Pneumocystis Jirovecii Pneumonia. EJIFCC. 1 oct 2025;36(3):407‑11. PubMed PMID: 41098223; PubMed Central PMCID: PMC12519627.

33.	Nuñez-Nuñez ME, Valenzuela-Vazquez L, Bustamante-Ogando JC, Bayardo-Gutierrez B, Lona-Reyes JC, Estrada-Arce EV, et al. A novel pathogenic *RFXANK* variant causing MHC class II deficiency in two unrelated patients from Mexico. Hum Immunol. 1 mars 2026;87(3):111655. doi:10.1016/j.humimm.2026.111655

34.	Haskologlu S, Aytekin C, Islamoglu C, Kostel Bal S, Baskın K, Erman B, et al. Clinical, immunological, treatment characteristics, and outcomes in 22 patients with major histocompatibility complex class II deficiency. Front Immunol. 17:1811502. doi:10.3389/fimmu.2026.1811502 PubMed PMID: 42099591; PubMed Central PMCID: PMC13143760.
