# Supplementary material for: MHC-II deficiency in a large Algerian cohort: Clinical, immunological, and genetic characterization
Source: J Hum Immun. 2026 Oct 30;3(1):e20260162. doi: 10.70962/jhi.20260162 (PMC13721797; doi:10.70962/jhi.20260162)
Supplement: Supplementary file 5 [file jhi_20260162_tables5.docx]

**Table S5.** Published *RFXAP* variants reported in MHC class II deficiency

| **Mutation** | **Case number** | **Ethnicity** | **Author** | **Year of publication** | **Reference** |
| --- | --- | --- | --- | --- | --- |
| c.163C>T  p.Gln55Ter  (*formerly c.279C>G*) | 1  1  1 | Turkish  Turkish  Iranian | Villard et al  Fondaneche et al  Musavi Khorshidi et al | 1997  1998  2023 | [1]  [2]  [3] |
| c.368del p.Ser123ThrfsTer15  (*formerly c.484delG*) | 1  2  2  1 | Moroccan  Algerian (1)  Moroccan (1)  North African  Austrian | Durand et al  Villard et al  Fondaneche et al  Schmetterer et al | 1997  1997  1998  2010 | [4]  [1]  [2]  [5] |
| c.150_151insGCGGGGC  p.Pro51AlafsTer24  (*formerly c.151ins7*) | 2 | Druze | Fondaneche et al | 1998 | [2] |
| 75bp insertion in 5-UTR | 1  1 | Turkish  Turkish | Van Eggermond et al  Ünsal et al | 2008  2023 | [6]  [7] |
| c.219C>G  p.Tyr73Ter | 1 | Mexican | Gokturk et al | 2012 | [8] |
| c.323T>A  p.Leu108Ter | 1 | Mexican | Wang et al | 2014 | [9] |
| c.460_461insC p.Lys155GlnfsTer21 | 2 | Indian | Aluri et al | 2018 | [10] |
| c.709-1G>T | 1 | Indian | Aluri et al | 2018 | [10] |
| c.350del  p.His117ProfsTer21 | 1 | Iranian | Musavi Khorshidi et al | 2023 | [3] |
| c.467del  p.Gln156ArgfsTer28 | 1 | Iranian | Musavi Khorshidi et al | 2023 | [3] |
| c.721del  p.Ser241ValfsTer4 | 1 | Turkish | Gulec Koksal et al | 2024 | [11] |
| Exon 1 large deletion | 1 | Turkish | Gulec Koksal et al | 2024 | [11] |
| c.751C>T  p.Gln251Ter | 2 | Turkish | Haskologlu et al | 2026 | [12] |
| c.757C>T  p.Gln253Ter | 1 | Turkish | Haskologlu et al | 2026 | [12] |

**References**

1.	Villard J, Lisowska-Grospierre B, Elsen P van den, Fischer A, Reith W, Mach B. Mutation of RFXAP, a Regulator of MHC Class II Genes, in Primary MHC Class II Deficiency. N Engl J Med. 11 sept 1997;337(11):748‑53. doi:10.1056/NEJM199709113371104

2.	Fondaneche MC, Villard J, Wiszniewski W, Jouanguy E, Etzioni A, Le Deist F, et al. Genetic and Molecular Definition of Complementation Group D in MHC Class II Deficiency. Hum Mol Genet. 1 mai 1998;7(5):879‑85. doi:10.1093/hmg/7.5.879

3.	Mousavi Khorshidi MS, Seeleuthner Y, Chavoshzadeh Z, Behfar M, Hamidieh AA, Alimadadi H, et al. Clinical, Immunological, and Genetic Findings in Iranian Patients with MHC-II Deficiency: Confirmation of c.162delG RFXANK Founder Mutation in the Iranian Population. J Clin Immunol. 1 nov 2023;43(8):1941‑52. doi:10.1007/s10875-023-01562-z

4.	Durand B, Sperisen P, Emery P, Barras E, Zufferey M, Mach B, et al. RFXAP, a novel subunit of the RFX DNA binding complex is mutated in MHC class II deficiency. EMBO J. 3 mars 1997;16(5):1045‑55. doi:10.1093/emboj/16.5.1045 PubMed PMID: 9118943; PubMed Central PMCID: PMC1169704.

5.	Schmetterer KG, Seidel MG, Körmöczi U, Rottal A, Schwarz K, Matthes-Martin S, et al. Two newly diagnosed HLA class II-deficient patients identified by rapid vector-based complementation analysis reveal discoordinate invariant chain expression levels. Int Arch Allergy Immunol. 2010;152(4):390‑400. doi:10.1159/000288292 PubMed PMID: 20197681.

6.	van Eggermond MCJA, Tezcan I, Heemskerk MHM, van den Elsen PJ. Transcriptional silencing of *RFXAP* in MHC class II-deficiency. Mol Immunol. 1 mai 2008;45(10):2920‑8. doi:10.1016/j.molimm.2008.01.026

7.	Ünsal H, Caka C, Bildik HN, Esenboğa S, Kupesiz A, Kuşkonmaz B, et al. A large single-center cohort of bare lymphocyte syndrome: Immunological and genetic features in Turkey. Scand J Immunol. 2024;99(1):e13335. doi:10.1111/sji.13335

8.	Gokturk B, Artac H, van Eggermond MJ, van den Elsen P, Reisli İ. Type III bare lymphocyte syndrome associated with a novel RFXAP mutation: a case report. Int J Immunogenet. août 2012;39(4):362‑4. doi:10.1111/j.1744-313X.2012.01105.x PubMed PMID: 22390233.

9.	Wang JC, Ross L, Mahon LW, Owen R, Hemmat M, Wang BT, et al. Regions of homozygosity identified by oligonucleotide SNP arrays: evaluating the incidence and clinical utility. Eur J Hum Genet. mai 2015;23(5):663‑71. doi:10.1038/ejhg.2014.153 PubMed PMID: 25118026; PubMed Central PMCID: PMC4402629.

10.	Aluri J, Gupta M, Dalvi A, Mhatre S, Kulkarni M, Hule G, et al. Clinical, Immunological, and Molecular Findings in Five Patients with Major Histocompatibility Complex Class II Deficiency from India. Front Immunol. 2018;9:188. doi:10.3389/fimmu.2018.00188 PubMed PMID: 29527204; PubMed Central PMCID: PMC5829618.

11.	Gulec Koksal Z, Bilgic Eltan S, Topyildiz E, Sezer A, Keles S, Celebi Celik F, et al. MHC Class II Deficiency: Clinical, Immunological, and Genetic Insights in a Large Multicenter Cohort. J Allergy Clin Immunol Pract. 1 sept 2024;12(9):2490-2502.e6. doi:10.1016/j.jaip.2024.06.046

12.	Haskologlu S, Aytekin C, Islamoglu C, Kostel Bal S, Baskın K, Erman B, et al. Clinical, immunological, treatment characteristics, and outcomes in 22 patients with major histocompatibility complex class II deficiency. Front Immunol. 17:1811502. doi:10.3389/fimmu.2026.1811502 PubMed PMID: 42099591; PubMed Central PMCID: PMC13143760.
